# Elavl1 Impacts Osteogenic Differentiation and mRNA Levels of Genes Involved in ECM Organization

**DOI:** 10.3389/fcell.2021.606971

**Published:** 2021-02-04

**Authors:** Satya K. Kota, Zhu Wei Lim, Savithri B. Kota

**Affiliations:** ^1^Division of Bone and Mineral Research, Department of Oral Medicine, Infection and Immunity, Harvard School of Dental Medicine, Harvard University, Boston, MA, United States; ^2^Division of Nephrology, Beth Israel Deaconess Medical Center and Harvard Medical School, Boston, MA, United States

**Keywords:** RNA binding protein, mesenchmal stem cell, ECM organization, osteogenic differentiation, adenylate-uridylate-rich elements, RNA stability

## Abstract

Posttranscriptional gene regulation by Adenylate Uridylate (AU) rich element RNA binding protein, Elavl1 has been implicated in embryonic development as well as progenitor cell differentiation. Elavl1 binds to hundreds of cellular messenger RNAs predominantly through interactions with AU-rich elements (AREs) found in the untranslated regions (UTRs) and functions by regulating their stability. Biological functions of Elavl1 during osteogenic differentiation of bone marrow derived mesenchymal stem cells is not well-understood. Here we report that specific knockdown of nuclear localized Elavl1 by RNA interference in multipotent BMSCs led to increased osteogenic differentiation. Differential gene expression analysis following unbiased total RNA sequencing upon Elavl1 depletion during osteogenic differentiation of BMSCs showed increased levels of multiple mRNAs that are involved in extracellular matrix organization. We further show that many of these mRNAs contain Elavl1 binding consensus motifs that are preserved in their 3′ UTRs. RNA stability analyses indicated that depletion of Elavl1 prolongs the steady state RNA levels of several of these mRNAs. Together, our data points to Elavl1 mediated negative regulation of multiple genes involved in ECM organization that play a functional role in MSC osteogenic differentiation.

## Introduction

Several sequence non-specific RNA binding proteins play key roles during RNA biogenesis, processing, transport and stability (Lelli et al., 2012; Corbett, 2018; Hentze et al., 2018). Elavl1 (embryonic lethal-abnormal vision like 1) is one of the four highly conserved members of ELAV family proteins that play important roles in gene regulation with high affinities for U- and AU- rich sequence (ARE) containing RNAs (Simone and Keene, 2013). Elavl1 is predominantly localized in the nucleus but can shuttle between nucleus and cytoplasm under specific stimuli and contains an internal shuttling sequence that interacts with specific nuclear import and export proteins (Fan and Steitz, 1998a; Guttinger et al., 2004). Elavl1 shuttling has been shown to be dependent on certain stimuli including stress that increases the cytoplasmic localization of Elavl1 via posttranslational modifications (Gallouzi et al., 2001; Grammatikakis et al., 2017). Elavl1 has important roles in regulation of RNA stability in multiple tissues generally via interactions with AU rich elements in the 3′UTRs of messenger RNAs (Lebedeva et al., 2011; Mukherjee et al., 2011). Interactions of between ARE containing mRNAs in the nucleus and Elavl1 in general lead to stability by preventing access of these mRNAs to RNA binding proteins that recruit additional proteins involved in RNA decay pathway (Fan and Steitz, 1998b; Brennan and Steitz, 2001; Gallouzi et al., 2001; Wang et al., 2001). Genome-wide analysis of Elavl1 binding and RNA stability analyses have also revealed positive correlation between number of Elavl1 binding sites per transcript and their stability (Mukherjee et al., 2011). Loss of function mutations in *Elavl1* cause embryonic lethal phenotypes in fruit flies and mice (Campos et al., 1985; Katsanou et al., 2009). Elavl1 is indispensable in progenitor cells with functions in both survival and proliferation. In mouse, both prenatal as well post-natal deletion of *Elavl1* is lethal. Prenatal deletion of *Elavl1* leads to defects in both placental as well as embryonic development (Katsanou et al., 2009). In a tamoxifen induced postnatal Elavl1 deletion mouse model, atrophy of several tissues, including hematopoietic organs, intestinal villi and mortality in <2 weeks was observed (Ghosh et al., 2009). Several studies have also demonstrated important roles for Elavl1 in regulating the transcriptional output in adipocyte lineage and control of adipogenesis (Gantt et al., 2005; Li et al., 2019; Siang et al., 2020). Functional roles of many RNA binding proteins including Elavl1 in osteogenic differentiation are yet to be determined. In this study, using mouse bone marrow derived mesenchymal stem cells (BMSCs), we demonstrate that selective depletion of nuclear localized Elavl1 negatively regulates osteogenic differentiation, controls expression levels and stability of several AU rich element containing mRNAs associated with extracellular matrix organization.

## Materials and Methods

### Cell Lines and Treatments

W-20-17 (W-20) murine bone marrow stromal cells were described in Thies et al. (1992) and ST-2 cells were purchased from RIKEN Bioresource Research Center (RCB). ST-2 and W-20 cell lines were maintained in Dulbecco's modified Eagle's medium (Gibco) supplemented with 10% fetal bovine serum (Gibco). Osteoblast differentiation was induced by culturing the stromal cell lines in osteogenic differentiation medium (OM) containing 10 mMbeta-glycerol phosphate(sigma) and 100 μg/ml ascorbic acid for indicated time in days with medium changes every 48 h. Adipogenic differentiation was initiated by treating ST-2 and W-20 cells in presence of 1 uM Rosiglitazone with medium change every 72 h. For Actinomycin D treatment, W-20 cells were differentiated in the presence of osteogenic medium for 14 days and then treated with Actinomycin D, 1 ug/ml concentration (Sigma Aldrich). Cells were washed thoroughly with PBS and collected at indicated timepoints for total RNA extraction and subsequently for reverse transcription followed by real-time PCR quantification.

### Stable Elavl1 Knockdown

shRNA oligos were cloned in pSIREN-RetroQ (Clontech) with BamH1 and EcoR1 overhangs. Retroviral supernatants were collected after transfecting the shRNA constructs into Plat-E cells (Cell Biolabs). Two regions within mRNA sequence of Elavl1 were chosen for silencing. Elavl1 shRNA-1: 5′-CCC ACA AAT GTT AGA CCA ATT-3′ and Elavl1 shRNA-2: 5′-CGA GGT TGA ATC TGC AAA GCT-3′. Stable pools of control or Elavl1 shRNA transduced W-20 and ST-2 were maintained in 2.5 ug/ml puromycin (invivogen).

### Real Time PCR Quantification

Total RNA was prepared from cultured cells using RNeasy kit (Qiagen). DNA contamination was eliminated by DNaseI digestion. Complementary DNA was prepared using multiscript reverse transcription system (Applied biosystems). The reverse transcribed cDNA was subjected to qPCR using a Sybr green based detection system (Qiagen). Relative levels of transcripts were normalized to levels of housekeeping genes and quantified based on 2^−ΔΔCT^ method. A minimum of 2–5 independent biological replicates were analyzed. The primer sequences used will be available upon request.

### Alkaline Phosphatase Staining

W-20 or ST-2 cells were cultured in osteogenic differentiation medium (OM) with medium change every 48 h. On day 10 post incubation, cells were washed twice with PBS and fixed in 4% (v/v) paraformaldehyde (sigma) for 5 min. Cells were washed and incubated with alkaline phosphatase substrate solution (SigmaFast BCIP-NBT; Sigma Aldrich) for 10–20 min in dark. Cells were washed three times with wash buffer containing PBS and 0.05% Tween-20 to remove the substrate and immersed in distilled water and used for imaging under brightfield.

### Alizarin Red Staining

For Alizarin red staining, cells were cultured and differentiated as described above and on day 20 post culturing, cells were washed twice with PBS and fixed in 4% (v/v) paraformaldehyde (sigma) for 15 min. Cells were washed five times with deionized water and incubated with 2% alizarin red solution for 20 min and washed five times with distilled water to remove excess staining solution and imaged under brightfield. For quantitation, alizarin red was extracted with 10% acetic acid and after neutralization with 10% ammonium hydroxide, absorbance was read at 405 nm.

### Immunofluorescence and Western Blot Analysis

Cell lines (W-20, ST-2) and Elavl1 knockdown W-20 cells were grown on coverslips and differentiated for 10 days in the presence of adipogenic or osteogenic medium. Cells were fixed with 4% paraformaldehyde, followed by permeabilization with 1% Triton X-100. Cells were incubated for 2 h with anti Elavl1 antibody (Santacruz Biotechnology, SC-71290) and appropriate fluorescent-tagged secondary antibodies were used to detect antibody specific signals as previously described (Kota et al., 2018). For western blot analysis, total cell lysates were prepared using RIPA lysis buffer supplemented with protease inhibitor cocktail. Equal amounts of protein samples were separated on a 4–12% polyacrylamide gel (Bio-Rad), blotted onto polyvinylidene difluoride membrane, and probed with anti Elavl1 antibody (Santacruz Biotechnology, SC-71290, dilution,1:250) and anti Actin antibody (Abcam, dilution, 1:2000). Appropriate horseradish peroxidase conjugated secondary antibodies were used and signals were developed using chemiluminescent substrates (Amersham ECL prime detection reagents).

### RNA Sequencing Analysis

RNA sequencing was performed on total RNA isolated from W-20 control shRNA or Elavl1KD cells cultured in the presence of osteogenic differentiation medium for 10 days and total RNA from two independent experiments was subjected to sequencing. Library preparation and sequencing were performed by the Biopolymers Facility at Harvard medical school. Data was processed using a standard RNA -seq pipeline that used STAR aligner to align the reads to mm10. Cufflinks suite and cuffdiff2 was used to calculate differential expression (Trapnell et al., 2012, 2013). List of genes that were 1.5-fold up or downregulated (log2FC) in Elavl1KD samples with a q value (FDR corrected *P*-value of the test statistic) of <0.05 were chosen for further analysis. Results of Reactome pathway term (Fabregat et al., 2018) output via enrichr (Chen et al., 2013) for the up- or down-regulated genes were sorted based on *P*-values < 0.01.

### Statistical Analysis

All comparisons were performed with a two-tailed unpaired Student's *t*-test. The data are expressed as mean ± SEM. For data that are normalized, data analyses were performed following the method described by Valcu and Valcu (Valcu and Valcu, 2011) and essentially as described in Kota et al. (2018). Briefly, values for control and experimental samples were divided by the mean of the control sample, which conserves the distribution and the relative variance of the samples, allowing the subsequent use of a *t*-test: mean (k ^*^ X) = k ^*^ mean(X); std error (k ^*^ X) = k ^*^ std error(X), where X is the sample and k = 1/mean of the control and is a normalization factor. p values are indicated by ^*^ for *p* < 0.05; ^**^ for *p* < 0.01; ^***^ for *p* < 0.001.

## Results and Discussion

**Figure 1:**
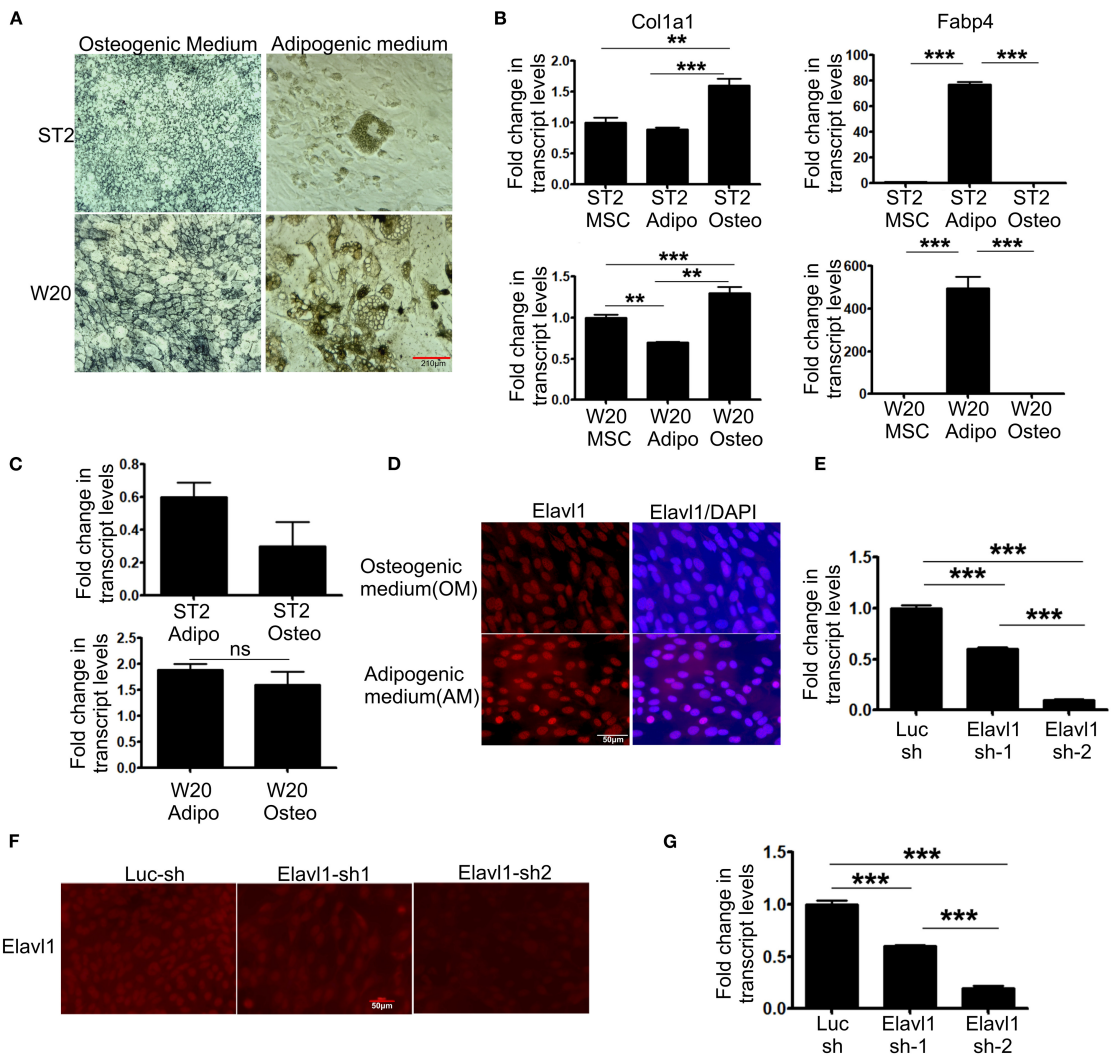
Differentiation potential of bone marrow derived mesenchymal stromal cells, ST2 and W-20 into Osteogenic, Adipogenic lineages; gene expression, localization of Elavl1 and silencing of Elavl1 mRNA by RNAi. Differentiation potential of W-20 and ST-2 MSCs used in this study was evaluated by culturing the respective cells in osteogenic (OM) or adipogenic medium (AM) for 2 weeks. Differentiation was ascertained by **(A)** staining cells for alkaline phosphatase, marker for osteogenic potential (left panel, top, ST2; bottom, W-20) or Oil red O, marker for adipogenic potential (right panel, top, ST2; bottom, W-20). Scale bar 210μm. **(B)** gene expression analysis of Col1a1 (osteo-left panel) and Fabp4 (adipo-right panel) **(C)** qRT-PCR analyses of Elavl1 expression during adipogenic or osteogenic differentiation in ST2 (top) and W20 (bottom) MSCs. **(D)** Nuclear localization of Elavl1 protein (red) in ST2 cells upon differentiation in the presence of osteogenic and adipogenic medium. Nuclei were stained with DAPI. Scale bar 50 μm Cellular levels of Elavl1 were depleted in W-20 by using two shRNAs targeting two distinct regions within the messenger RNA by retroviral RNAi. Loss of Elavl1 expression upon shRNA mediated knockdown compared to control shRNA was analyzed at both mRNA and protein levels in MSCs by RT-qPCR **(E)**, immunofluorescence [**(F)**, Scale bar 210 μm] and during osteogenic differentiation **(G)**. (***P < 0.001; **P < 0.05).

To evaluate the expression dynamics of Elavl1 during osteogenic differentiation of mesenchymal stem cells, we utilized two BMSCs, W-20, and ST-2. Both these mesenchymal stem cell lines showed efficient differentiation potential upon osteogenic and adipogenic stimuli as analyzed by alkaline phosphatase and oil red O staining respectively and key marker gene expression of osteo (*Col1a1*), adipogenic (*Fabp4*) differentiation (Figures 1A,B). We utilized these cells to analyze the expression dynamics of Elavl1 using RT-qPCR. At day 14, messenger RNA (mRNA) levels of Elavl1 were not significantly different between osteogenic and adipogenic differentiation conditions in both W-20 and ST2 cells (Figure 1C). As Elavl1 protein can exhibit nucleo-cytoplasmic shuttling under certain stimuli, we evaluated the localization of Elavl1 during osteogenic and adipogenic differentiation. Immunofluorescence analyses revealed that in both ST-2 and W-20 MSCs, Elavl1 were predominantly nuclear during osteogenic and adipogenic differentiation (Figure 1D, Supplementary Figure 1). To understand the biological functions of Elavl1 specifically in osteogenic differentiation of BMSCs, we performed stable RNA interference using two unique shRNAs targeting two distinct regions within Elavl1. Elavl1 shRNA-1 and shRNA-2 reduced mature Elavl1 transcript levels by 40 and 90%, respectively in W-20 MSCs that was further validated by reductions at the protein levels with negligible to no nuclear staining in Elavl1sh2 stable knockdown cells (Figures 1E,F). Stable reduction of Elavl1 transcript levels were further confirmed upon osteogenic differentiation of W-20 (Figure 1G) and in ST-2 MSCs (Supplementary Figures 2A,B). As silencing efficiency of shRNA-2 was superior than shRNA-1, Elavl1 shRNA-2 (Elavl1 KD) was used for further characterization of Elavl1 depletion effects on osteogenic differentiation. Western blot analysis was further employed to assess the silencing efficiency of Elavl1 KD both in undifferentiated and upon osteogenic differentiation (Supplementary Figure 3).

**Figure 2:**
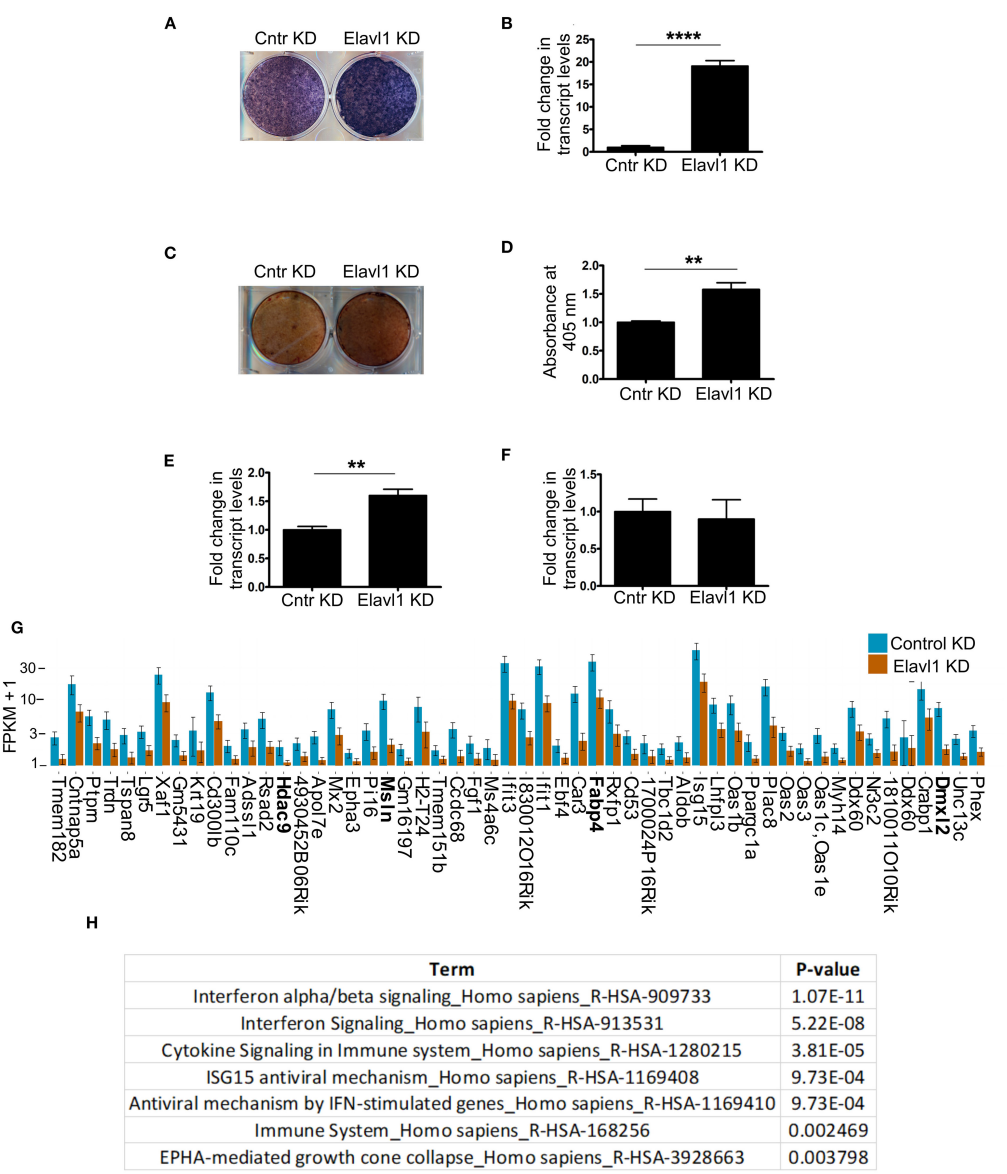
Effect of Elavl1 knockdown on osteogenic differentiation potential of mesenchymal stromal cells (MSCs) and RNA-sequencing analysis of W-20 MSCs undergoing osteogenic differentiation. Stable knockdown of Elavl1 during differentiation of W-20 cells into osteoblast lineage led to increased alkaline phosphatase activity as assessed by staining on Day 10 (a representative image is shown, *N* = 3) **(A)**, increased Alpl mRNA levels by RT-qPCR, (****, *P* < 0.0001) **(B)**. Increased mineralization was seen by Alizarin red staining upon Elavl1 knockdown **(C)**, as quantified in **(D)** (**P < 0.01). Elavl1 knockdown increased transcript levels of RunX2 mRNA levels (***P < 0.001) **(E)** but not Osx mRNA levels as assessed by qRT-PCR analysis **(F)**. Total RNA was collected from Luc shRNA (Control KD) and Elavl1 shRNA-2 (Elavl1 KD) W-20 MSC cells undergoing differentiation in presence osteogenic medium (OM) at Day 10 and RNA sequencing was performed. Bar plot of FPKM expression values of genes downregulated more than 1.5-fold (log2FC, *q* < 0.05) upon Elavl1 knockdown in W-20 cells following differentiation into osteoblasts. Genes whose expression is validated by RT-qPCR (Supplementary Figure 6) are highlighted **(G)**. Reactome pathway term analysis (*p* < 0.01) of RNA-seq data revealed that genes involved in interferon pathway to be overrepresented and downmodulated by Elavl1 knockdown in W-20 MSCs during osteogenic differentiation **(H)**.

Elavl1 has been shown to both positively and negatively regulate mRNA stability, thereby affecting cellular physiology in multiple cell types and tissues. To understand effects of Elavl1 silencing on osteogenic differentiation, we cultured MSCs stably expressing Elavl1 shRNA or control shRNA in the presence of osteogenic induction medium (OM) containing 10 mM beta-glycerol phosphate and 100 μg/ml ascorbic acid. Alkaline phosphatase and alizarin red staining of W-20 MSCs cultured in OM in the presence of control shRNA (Cntr KD) or Elavl1 shRNA-2 (Elavl1 KD) on Day 10 showed increases in tissue non-specific alkaline phosphatase (TNAP) enzyme activity as well as transcript levels upon Elavl1 depletion (Figures 2A,B). Increased TNAP enzyme activity was also seen in ST-2 cells upon Elavl1 knockdown during osteogenic differentiation (Supplementary Figure 4). Increased Alizarin red staining was also observed in Elavl1 knockdown cells compared to control cells on Day 20 in the presence of OM (Figures 2C,D). Interestingly, analysis of transcript levels of osteogenic lineage markers, Runt-related transcription factor 2 (Runx2) and Osterix (Osx) revealed significant increase only in mRNA levels of Runx2 (Figure 2E), whereas Osx mRNA levels remained unchanged upon Elavl1 knockdown (Figure 2F). To understand the genome-wide molecular changes that occur upon Elavl1 RNA interference upon osteoblast differentiation, total RNA isolated from W-20 cells stably expressing either control shRNA and Elavl1 shRNA following osteoblast differentiation (Day 10) were subjected to bulk RNA sequencing. Computation of RNA-seq data by principal component and dendrogram analyses revealed separation of samples predominantly based on biological variation (Supplementary Figures 5, 6). Transcripts levels that were altered more than 1.5-fold up or down (log2FC, *q* < 0.05) upon Elavl1 knockdown were deduced by differential gene expression analysis using Cuffdiff. Majority of the genes that were significantly downregulated 1.5-fold or more (log2FC) (Supplementary Table 1) upon Elavl1 knockdown included multiple genes involved in interferon signaling (Mx2, Ifit1, Ifit3, Oas2, Oas3) as deduced by reactome pathway analysis. Few other genes previously shown to be involved in the process of adipogenesis (e.g., Fabp4) were also significantly down-regulated upon Elavl1 silencing (Figures 2G,H). Besides Fabp4, genes whose transcripts were 1.5-fold or more (log2FC) downregulated were randomly chosen (Hdac9, Msln, Dmxl2) from the RNA-seq dataset and were further confirmed by RT-qPCR analysis (Supplementary Figure 7).

**Figure 3:**
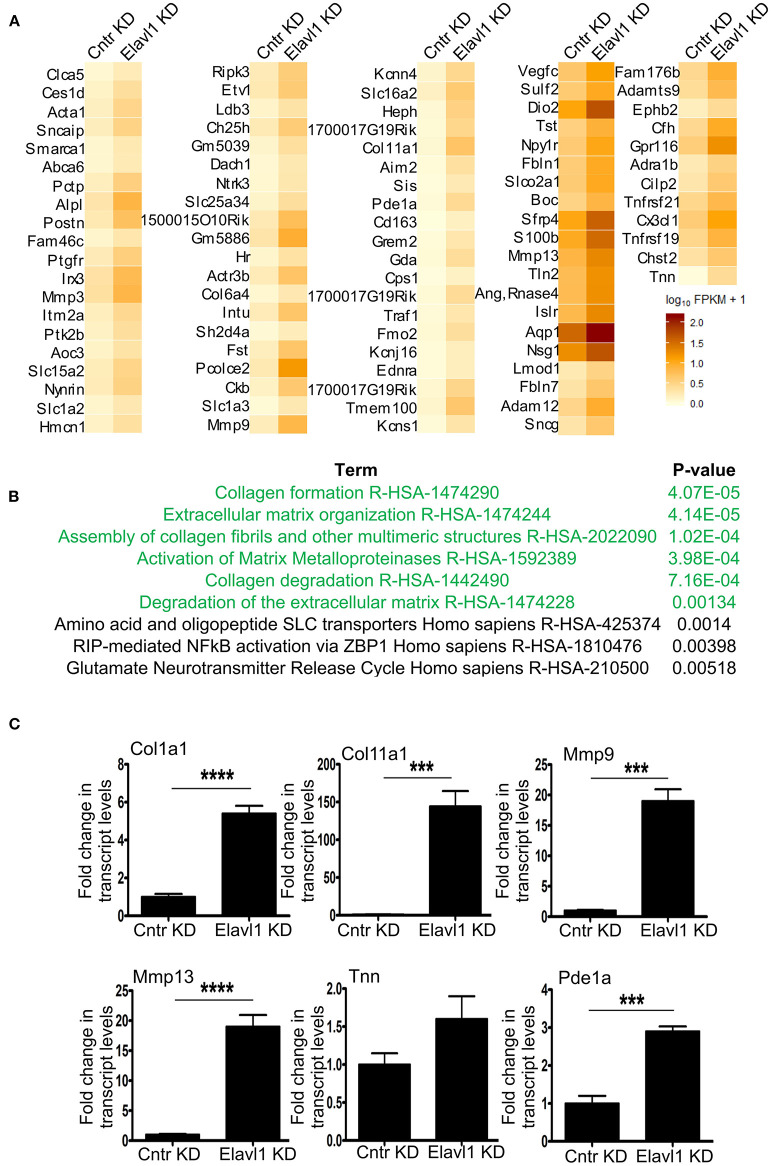
Elavl1 knockdown upregulates genes involved in extracellular matrix organization in W-20 MSCs undergoing osteogenic differentiation. **(A)** Heatmap of genes upregulated 1.5-fold or more (log2FC, *q* < 0.005) upon Elavl1 KD in W-20 cells following differentiation into osteoblasts. **(B)** Reactome pathway term analysis(*p* < 0.005) of RNA-seq data revealed that genes involved in collagen biosynthesis and extracellular matrix organization (ECM) to be overrepresented in the upregulated gene set upon Elavl1 knockdown in W-20 MSCs during osteogenic differentiation. **(C)** Validation of RNA-sequencing data of randomly chosen, significantly upregulated genes including that play roles in collagen pathway (Col1a1, Col11a1) Matrix metalloproteinases(Mmp9, Mmp13), and others (Tnn, Pde1a) upon Elavl1 KD during osteogenic differentiation by using RT-qPCR (***P < 0.001; ****P < 0.0001).

Interestingly, Elavl1 depletion by RNAi led to significant upregulation of several mRNAs that are involved in extracellular matrix regulation that might play a role in osteogenic differentiation (Figure 3A). Gene set enrichment analysis using reactome pathway further revealed that extracellular matrix organization as the top significantly enriched term in the upregulated gene list (1.5 fold and above) following Elavl1 RNAi (Figure 3B). Validation of RNA-sequencing data was performed by RT-qPCR analysis on randomly chosen set of genes that were upregulated more than 1.5-fold or more upon Elavl1 knockdown and with a statistical significance of < 0.05 (Supplementary Table 2). This included the genes that were known to modulate extracellular matrix such as Collagen family (Col1a1, Col11a1), Matrix metalloproteinase gene family (Mmp3, Mmp13), as well as TNN and Pde1a (Figure 3C). Consistent with RNA sequencing results, W-20 cells undergoing osteogenic differentiation had significant increase in transcript levels of these genes upon Elavl1 knockdown. RT-qPCR analyses of ECM genes were also performed in ST-2 cells differentiated in osteogenic medium which revealed similar increase in transcript levels (Supplementary Figure 8).

**Figure 4:**
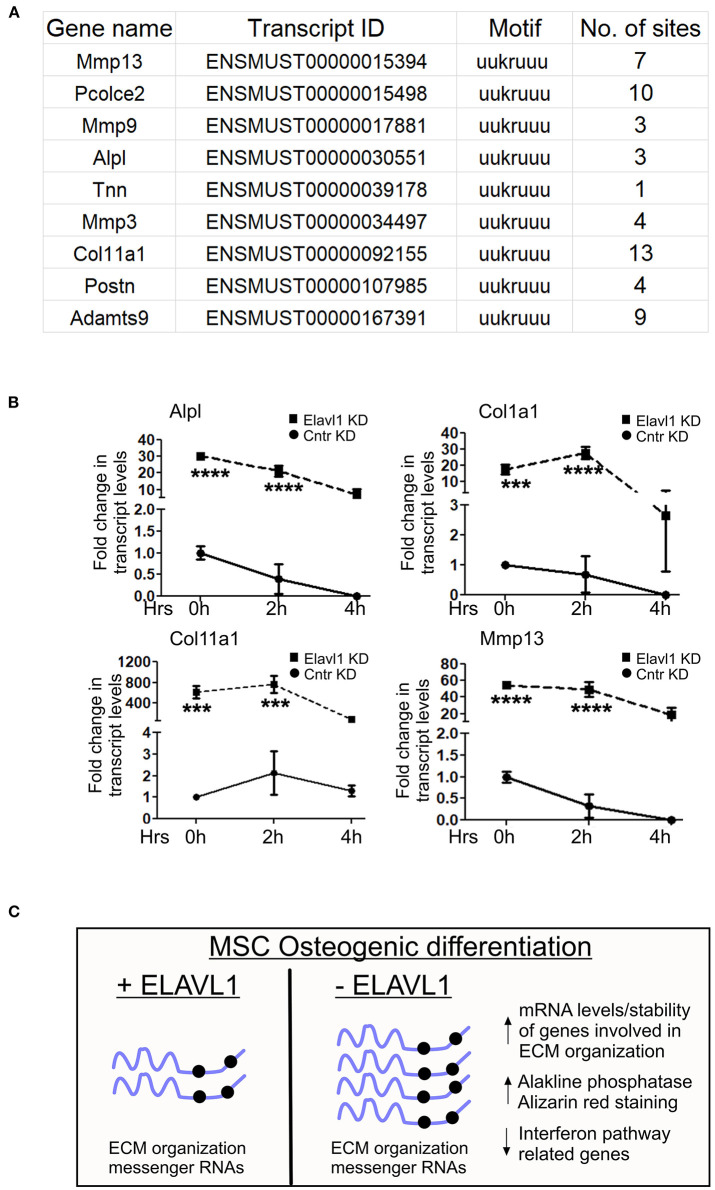
Regulation of stability of ECM mRNAs by Elavl1. Elalvl1 protein binds to AU rich elements in the 3′ untranslated regions (3 UTR) regions of mRNAs and regulates their stability. **(A)** Sequence analysis of 3′UTR regions of ECM mRNAs that were upregulated during osteogenic differentiation revealed presence of multiple Elavl1 consensus binding motifs. **(B)** RT-qPCR analysis of total RNA from Act-D treated W-20 MSCs 14 days post osteogenic differentiation collected after 0, 2, and 4 h shows Elavl1 negatively regulates stability of ECM gene mRNAs (Alpl, Col1a1, Col11a1, Mmp13) (***P < 0.001; ****P < 0.0001). **(C)** Model showing summary of finding described in this study.

Elavl1 is an RNA binding protein that predominantly binds to U or AU rich UTR regions of mRNAs and regulates their stability. We determined whether mRNAs that are upregulated upon Elavl1 knockdown indeed have Elavl1 binding AU rich elements (AREs). We analyzed 3′ UTR sequences of mRNAs corresponding to genes that were upregulated more than 1.5-fold and are implicated in extracellular matrix organization (Supplementary Text in Supplementary Material). In all of these mRNAs, more than one Elavl1 consensus ARE element was identified (Figure 4A). Next, to understand the effect of Elavl1 knockdown on the stability of these mRNAs, we have performed RNA stability assay using Actinomycin D that inhibits active RNA polymerase II transcription. Elavl1 stable knockdown W-20 MSCs were differentiated in the presence of osteogenic medium for 14 days and on day 14 cells were treated with ActD or vehicle and cells were collected at 0, 2, and 4 h time points. Upon ActD treatment, at 4 h time point, ECM mRNAs levels were very low in control shRNA cells in comparison with Elavl1KD cells, indicating possible negative regulation of these mRNAs by Elavl1 (Figure 4B). Next, we analyzed the presence of ARE elements in 3′ UTRs of ten ECM mRNAs that were shown to be up-regulated in the RNA-sequencing data upon Elavl1 knockdown and compared them with number of ARE elements in 3′ UTR regions of ten randomly chosen mRNAs that were downregulated 1.5-fold or more. In this select dataset, there were twice the number of AREs in the downregulated mRNAs compared to upregulated mRNAs (Supplementary Text in Supplementary Material). These results indicate that Elavl1 mediates negative regulation of certain ECM pathway genes and was confirmed using two mesenchymal stem cell lines (Figure 4C).

Elavl1 is a conserved RNA binding protein with high affinities for U or AU rich motifs present generally in 3′ UTR regions of protein coding mRNAs and performs key functions in gene regulation in multiple tissues. So far, the biological role and relevance of Elavl1 during osteogenic differentiation of BMSCs are not understood. In this study, we have demonstrated that Elavl1 depletion in BMSCs resulted in increased osteogenesis and positively correlated with increased ECM gene mRNA expression in two different MSC lines. Analysis of RNA-seq data from Elavl1 knockdown osteogenic cells led to identification of several mRNAs that were upregulated 1.5-fold or more and that functions in extracellular matrix organization. These mRNAs included matrix metallo-proteinase family (Mmp3, Mmp9, and Mmp13) as well as collagen family (Col1a1, Col6a4, Col11a1, Pcolce2) etc. Consistent with our results, HuR (Elavl1) knockdown has been previously shown to increase mRNA levels of Mmp3, Mmp13 (McDermott et al., 2016; Pan et al., 2019) in nucleus pulposus cells and chondrocytes. Similarly, upon HuR silencing, collagen mRNA levels were differentially regulated depending on the cell type (Ge et al., 2016; Pan et al., 2019). We have previously shown that IFN gene responses were significantly decreased and inversely correlated with osteogenic differentiation (Kota et al., 2018). Upon stable knockdown of Elavl1, mRNA levels of several genes that belong to interferon signaling pathway were significantly downregulated consistent with previously described roles in stability of IFN-B mRNA and type I interferon response (Herdy et al., 2015). In contrast to the well-characterized functions of Elavl1 in mRNA stability via interactions with ARE elements in mRNAs, several recent studies also point toward Elavl1 roles in mRNA destabilization. Cammas et al. (2014) have shown that Elavl1 recruitment of mRNA decay factors such as KSRP as well as ribonuclease PARN negatively regulates nucleophosmin (NPM) mRNA levels and thereby impacts early stages of myogenesis. Similar negative regulation of TIN2 mRNA levels by HuR controls replicative senescence associated ROS levels (Lee et al., 2018). All these studies point to important context specific mRNA destabilizing roles for Elavl1 in mouse and human cells. In our study, similar effect of Elavl1 depletion on stability of ECM gene expression was observed. Recent studies demonstrated key and deterministic roles for Elavl1 during adipocyte differentiation via control of specific mRNAs and positively modulating their stability (Li et al., 2019; Siang et al., 2020). Though it is not clear what determines the choice between stability and decay upon Elavl1 interactions with mRNAs, we found a correlation between the number of ARE elements in 3'UTRs of mRNA and destabilizing effect of Elavl1. Two-fold more ARE elements were seen in the 3' UTR of select set of mRNAs belonging to adipocyte regulation and interferon pathway response that were downregulated upon Elavl1 depletion compared to mRNAs belonging to ECM pathways that were upregulated. Previous genome-wide characterizations also have shown a link between number of Elavl1 binding sites in mRNAs with stability in human cell lines (Mukherjee et al., 2011). Whether similar correlation exist between the number of consensus motifs and stability of mRNAs in Elavl1 dependent manner and associated protein factors needs further study. Together, our results demonstrate effect of Elavl1 on osteogenic differentiation of mouse mesenchymal stem cells and ECM gene expression levels. Further studies are required to assess potential therapeutic benefits of modulating of Elavl1 levels in adult bone repair/remodeling.

## Data Availability Statement

The sequencing data generated in this study is available at NCBI (accession number: GSE158260).

## Author Contributions

SKK conceived the study and designed experiments. SKK, ZL, and SBK performed experiments and data analyses. SKK wrote the manuscript with input from other authors. All authors contributed to the article and approved the submitted version.

## Conflict of Interest

The authors declare that the research was conducted in the absence of any commercial or financial relationships that could be construed as a potential conflict of interest.

## Supplementary Material

Supplementary Figure 1 Nuclear localization of Elavl1 protein (red) in W-20 cells upon differentiation in the presence of osteogenic and adipogenic medium. Nuclei were stained with DAPI. Scale bar 50 μm.

Supplementary Figure 2 Cellular levels of Elavl1were depleted in ST-2 cells by using two shRNAs targeting two distinct regions within the messenger RNA by retroviral RNAi. Loss of Elavl1 expression upon shRNA mediated knockdown compared to control shRNA was analyzed by RT-qPCR in MSCs (left panel S2A, Day 0) as well as upon culture in osteogenic differentiation medium for 2 weeks (S2B, Day 14).

Supplementary Figure 3 Elavl1 protein levels were evaluated by Western blot analysis in stable pools of W-20 MSCs transduced with control shRNA (control KD) or Elavl1 shRNA2 (Elavl1 KD).

Supplementary Figure 4 Stable knockdown of Elavl1 during differentiation of ST-2 cells into osteoblast lineage in presence of 100ng/ml BMP2 led to increased alkaline phosphatase activity as assessed by staining at Day 10.

Supplementary Figure 5 Dendrogram obtained from VST transformed RNA-seq count data clustered replicates and separated based on biological variation.

Supplementary Figure 6 Principal component analysis of RNA sequencing data for W-20 cells stably knockdown for Elavl1 or control shRNA knockdown differentiating in osteogenic medium. 88.3 and 7.35% variance are explained by principal component 1 (PC1) and principal component 2(PC2). Red dots are Elavl1KD treated replicate samples and blue dots are Luciferase shRNA treated samples. First principal component, PC1 separated samples based expectedly on biological variation.

Supplementary Figure 7 Validation of RNA-sequencing data of randomly chosen genes that were significantly downregulated (Hdac9, Msln, Fabp4 and Dmxl2) upon Elavl1 KD in W-20 MSCs during osteogenic differentiation by using RT-qPCR (^***^*P* < 0.001; ^****^*P* < 0.0001).

Supplementary Figure 8 Gene expression analysis in ST-2 cells of mRNAs involved extracellular matrix organization (Col1a1, Col11a1, Pcolce2, Tnn) upon Elavl1 KD and following differentiation in presence of osteogenic medium by using RT-qPCR.

Supplementary Table 1 List of genes downmodulated 1.5-fold or less (log2FC) following the loss of Elavl1 W-20 cells undergoing osteogenic differentiation as deduced from RNA-sequencing analysis.

Supplementary Table 2 List of genes upregulated 1.5-fold or more (log2FC) following the loss of Elavl1 W-20 cells and undergoing osteogenic differentiation as deduced from RNA-sequencing analysis.

Supplementary Text High stringency HuR consensus motif(uukruuu)of select up-regulated and down-regulated mRNAs including those genes involved in ECM regulation and interferon response pathway were deduced using RBPmap (Paz et al., 2014).
