# Supplementary material for: Elavl1 Impacts Osteogenic Differentiation and mRNA Levels of Genes Involved in ECM Organization
Source: Front Cell Dev Biol. 2021 Feb 4;9:606971. doi: 10.3389/fcell.2021.606971 (PMC7889968; doi:10.3389/fcell.2021.606971)
Supplement: Supplementary Figure 1 — Nuclear localization of Elavl1 protein (red) in W-20 cells upon differentiation in the presence of osteogenic and adipogenic medium. Nuclei were stained with DAPI. Scale bar 50 μm. [file Data_Sheet_1.pdf]

Fig-S1

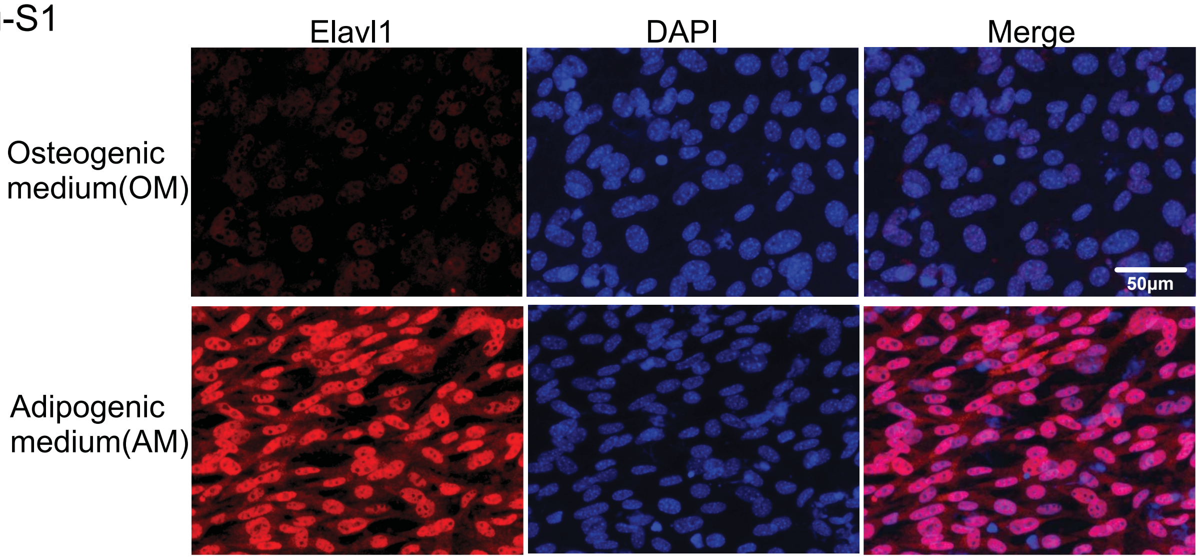

Fig-S2

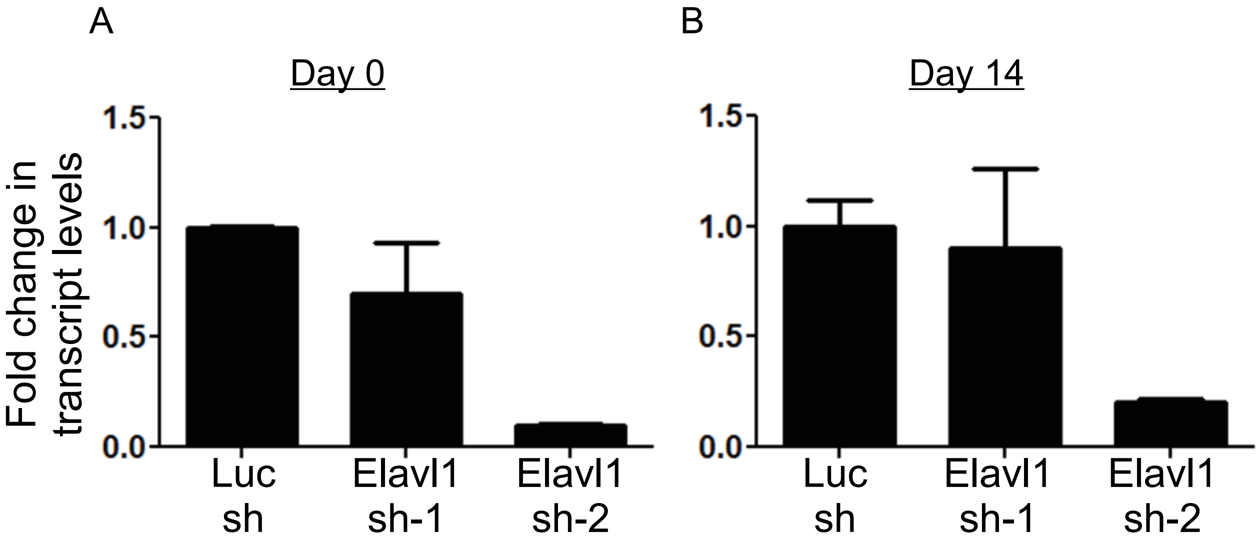

Fig-S3

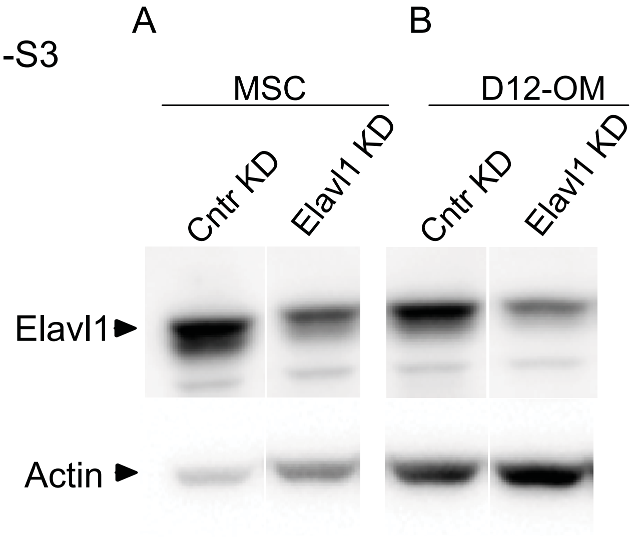

Fig-S4

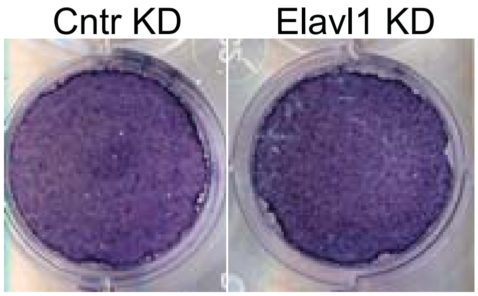

Fig-S5

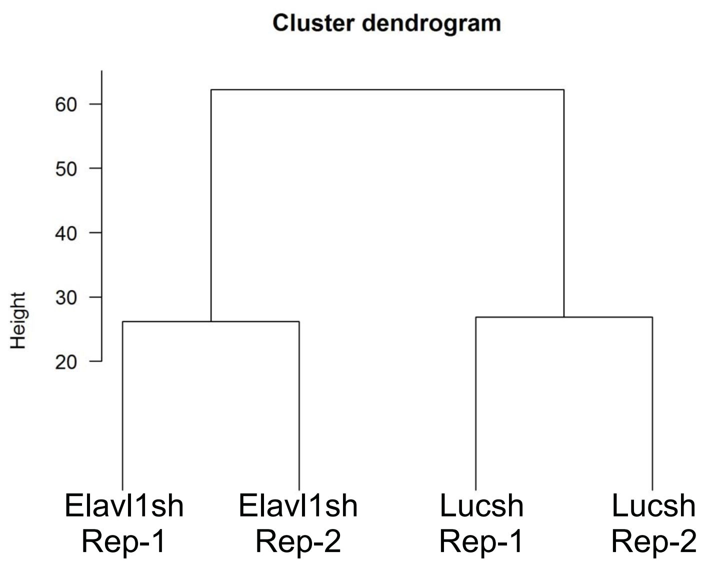
Cluster dendrogram
Height
60
50
40
30
20
Elavl1sh Rep-1
Elavl1sh Rep-2
Lucsh Rep-1
Lucsh Rep-2

Fig-S6

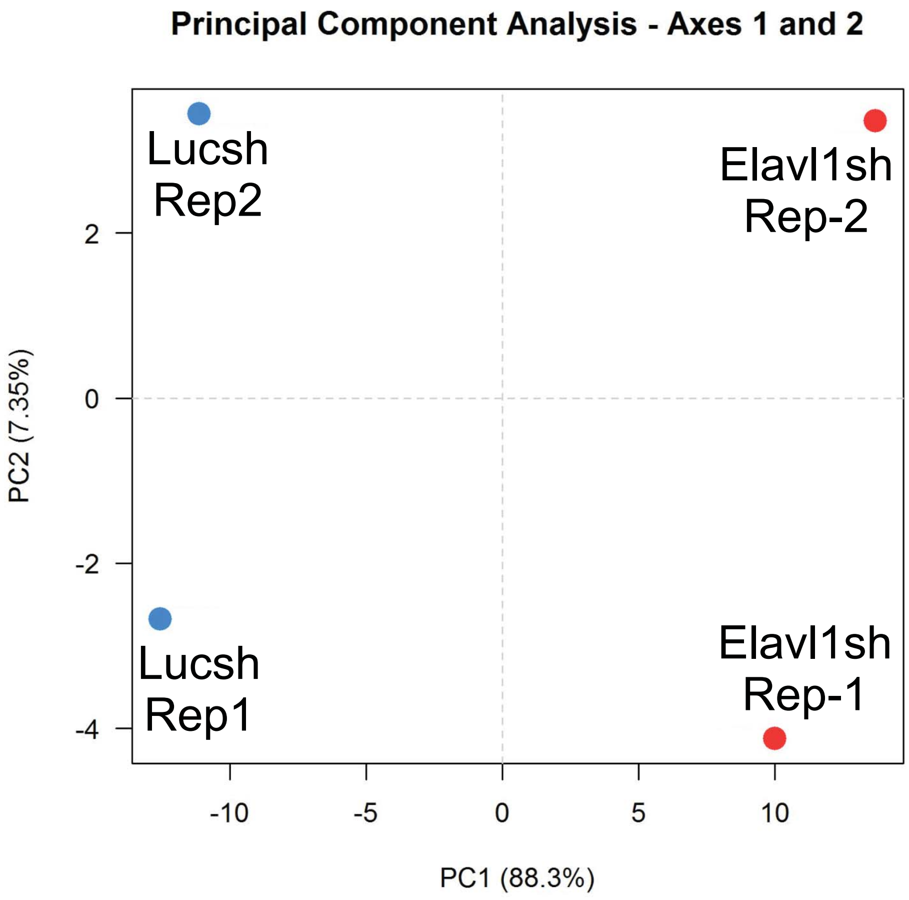
Principal Component Analysis - Axes 1 and 2
Lucsh Rep2
Elavl1sh Rep-2
Lucsh Rep1
Elavl1sh Rep-1
2
0
-2
-4
-10
-5
0
5
10
PC2 (7.35%)
PC1 (88.3%)

Fig-S7

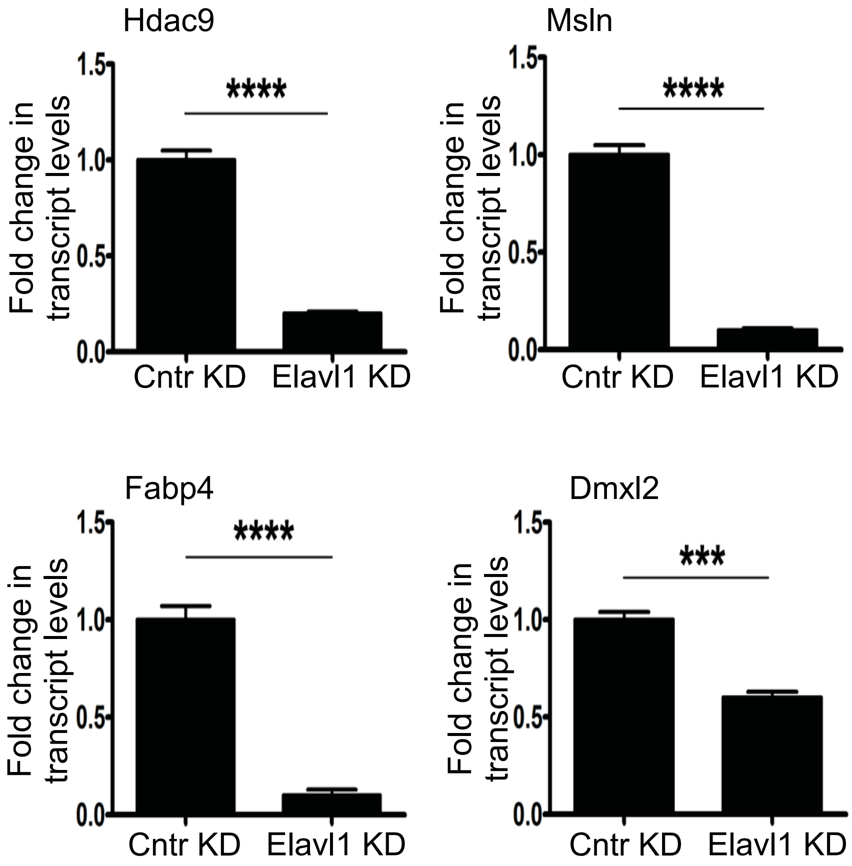

Hdac9
Msln
Fabp4
Dmxl2
Fold change in transcript levels
1.5
1.0
0.5
0.0
****
***
Cntr KD
Elavl1 KD

Fig-S8

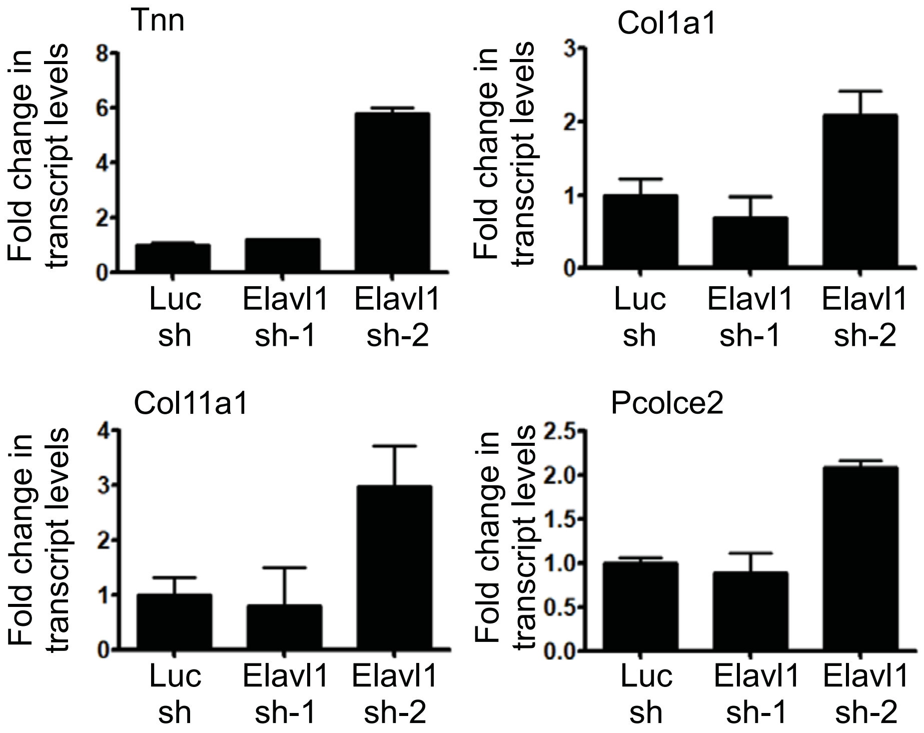

Tnn
Col1a1
Col11a1
Pcolce2
Fold change in transcript levels
Luc sh
Elavl1 sh-1
Elavl1 sh-2

Supplementary Table-1:
Top down-regulated genes

gene_id	gene	log2(fold_change)	q_value
XLOC_003803	Xaf1	-1.50407	0.002987
XLOC_028575	Ddx60	-1.50884	0.002987
XLOC_006125	Adssl1	-1.51212	0.002987
XLOC_028675	Nr3c2	-1.53831	0.002987
XLOC_021891	Lhfpl3	-1.53915	0.002987
XLOC_012699	Tmem151b	-1.54714	0.019822
XLOC_018652	Rxfp1	-1.55766	0.002987
XLOC_000351	Cntnap5a	-1.57891	0.002987
XLOC_020181	1700024P16Rik	-1.60015	0.038528
XLOC_012627	H2-T24	-1.61279	0.005485
XLOC_030374	Crabp1	-1.61692	0.002987
XLOC_021347	Isg15	-1.63637	0.002987
XLOC_023043	Oas2	-1.64219	0.002987
XLOC_008577	4930452B06Rik	-1.67254	0.002987
XLOC_002888	Lgr5	-1.67786	0.002987
XLOC_005171	Cd300lb	-1.67821	0.002987
XLOC_022316	Oas1b	-1.68893	0.002987
XLOC_010957	Mx2	-1.70583	0.002987
XLOC_015988	Ebf4	-1.76212	0.03737
XLOC_005034	Krt19	-1.79798	0.019822
XLOC_004500	Gm5431	-1.81652	0.002987
XLOC_011277	Epha3	-1.82973	0.033563
XLOC_018910	Cd53	-1.83323	0.002987
XLOC_014505	I830012O16Rik	-1.84888	0.002987
XLOC_020750	Tbc1d2	-1.88429	0.002987
XLOC_000907	Ptprn	-1.90934	0.002987
XLOC_018493	Fabp4	-1.91516	0.002987
XLOC_020760	Aldob	-1.92484	0.013489
XLOC_032944	Phex	-1.96635	0.002987
XLOC_031177	Unc13c	-1.97056	0.002987
XLOC_014506	Ifit1	-1.97643	0.002987
XLOC_014504	Ifit3	-1.98369	0.002987
XLOC_005769	Fam110c	-1.99885	0.013489
XLOC_011912	Pi16	-2.0003	0.002987
XLOC_013825	Fgf1	-2.02461	0.027309
XLOC_027252	Myh14	-2.10786	0.002987
XLOC_006198	Rsad2	-2.13144	0.002987
XLOC_012506	Gm16197	-2.15084	0.027309
XLOC_014806	Ms4a6c	-2.22017	0.033563
XLOC_022741	Ppargc1a	-2.25251	0.002987
XLOC_022895	Plac8	-2.3	0.002987
XLOC_023044	Oas3	-2.34488	0.002987
XLOC_001957	Trdn	-2.39016	0.002987
XLOC_023045	Oas1c,Oas1e	-2.46297	0.002987
XLOC_002334	Tspan8	-2.52002	0.002987
XLOC_013621	Cd74	-2.55138	0.011681

XLOC_013663	Ccdc68	-2.71071	0.002987
XLOC_000092	Tmem182	-2.73496	0.002987
XLOC_029079	1810011O10Rik	-2.75957	0.002987
XLOC_017910	Car3	-3.03859	0.002987
XLOC_031067	Dmxl2	-3.03914	0.002987
XLOC_012460	Msln	-3.0538	0.002987
XLOC_009473	Apol7e	-3.15709	0.002987
XLOC_006223	Hdac9	-3.20393	0.002987

Supplementary Table-2:
Top up-regulated genes

XLOC_008966	Dach1	2.01755	0.00298705
XLOC_027858	Adam12	2.03034	0.00298705
XLOC_008464	Hr	2.03725	0.00298705
XLOC_014907	Ch25h	2.04128	0.00548517
XLOC_029497	Acta1	2.04431	0.0134887
XLOC_020327	Fam176b	2.08435	0.00298705
XLOC_024029	Aqp1	2.08781	0.00298705
XLOC_021230	Slc25a34	2.13596	0.00775193
XLOC_006397	Gm5039	2.16392	0.00775193
XLOC_004896	Pctp	2.21604	0.00298705
XLOC_029331	Irx3	2.23131	0.00298705
XLOC_006400	Dio2	2.23902	0.00298705
XLOC_030086	Mmp3	2.27248	0.00298705
XLOC_001110	Cfh	2.31185	0.00298705
XLOC_017978	Intu	2.4754	0.00298705
XLOC_018002	Postn	2.49237	0.00298705
XLOC_021914	Actr3b	2.53741	0.00298705
XLOC_012086	Gpr116	2.55854	0.00298705
XLOC_025972	Kcnn4	2.60225	0.00298705
XLOC_009729	Slc1a3	2.66046	0.00298705
XLOC_021192	Alpl	2.67579	0.00298705
XLOC_029269	Ednra	2.68925	0.0396579
XLOC_000099	1500015O10Rik	2.69246	0.00298705
XLOC_007763	Fst	2.74151	0.00298705
XLOC_004225	Kcnj16	2.78226	0.00298705
XLOC_032812	Slc16a2	2.79575	0.00298705
XLOC_025059	Gm5886	2.95766	0.00298705
XLOC_016535	Traf1	2.99991	0.0490132
XLOC_006486	Ckb	3.08187	0.00775193
XLOC_001183	Fmo2	3.13312	0.00298705
XLOC_018557	1700017G19Rik	3.18102	0.00298705
XLOC_017225	Kcns1	3.22637	0.0408814
XLOC_000182	Cps1	3.24957	0.0151645
XLOC_016227	Mmp9	3.29602	0.00298705
XLOC_001275	Grem2	3.32809	0.00548517
XLOC_024310	Cd163	3.54006	0.0385275
XLOC_014862	Gda	3.56532	0.00298705
XLOC_018639	Sis	3.6044	0.00298705
XLOC_004006	Tmem100	3.70735	0.00298705
XLOC_000579	Aim2	3.80317	0.00298705
XLOC_016670	Pde1a	3.93254	0.00298705
XLOC_032236	Heph	3.9777	0.00298705
XLOC_030576	Pcolce2	4.13055	0.00298705
XLOC_018335	Col11a1	4.41148	0.00298705
XLOC_001163	Tnn	4.58353	0.00298705

Supplementary Text:

Down-regulated genes, Consensus motif (uukruuu)

>Oas3

AGTCTAGAGAGATCAGTGGTCACCATTGATAGAAAGTGACACCAGCCCTCAGCAAGTGATACTCAGAGTATCTGA
GTGTGTGTGTGTGTGTGTTGTATTTATCTGTATGTGTGTATTTGTGGTATGTCTGTGTGCCTATATGAGGGTGTGTC
TATGTGCGTGTCTGTGTATCTGTGGGTATCTATATGTGTCTGTATATATGTATGTGTGTGTGTGTGTGTGTGTAT
TCATGTATGTGTGTCTGTTTGTGTATAGTGTGTCTATAGGTGACTCTGTGTGTCTGTGTATCTGTGAGTATCTATAT
GTGTCTGTCTGTATGTAAATGTGTGTATGTATGTGTGTTCATATGTCTGTGTGTGTGTCTATATCTGTGTATCTTTGG
GTATCTATATGTGTCTATCTATATGTAAATGTATGTATGTACTTATGTTCATGTGTATCTGTGTGAATGTCTGTGTGT
TTATGTGTAGTGTATCTGTAAGTGTATCTGTATGTCTATAGATGTATTATGTCTTTGTGTGTCGACATGTCTGTGTGT
ATGTATGTTTGTATGTGTATGTTATATATGTATATATGCATGTATGTGCTTCCTCACACCATCTCCCTTCTGCCCACCT
GCCCACCCATAGCCCTCCCTTTCTTCCCACTGTTTACCCACCTGGTGGGGCTTCATTGACCTCAACCATGATCATCCC
GGTGTCCCTGACTCCCACACTAGACACCCTAGGAACCAGACATCTCTAGATCTTCTAGTCTGCTGTTCATCTACCAT
GGGCTCTGCCCCAACTTCCACAGCCCCACCCAGGAGTGCCTCAGCCCTGCCAAGAAGCCATACTCCTCCCTGGCAT
CTCTCTGCCCCTTGAGCCTGTGTATATCCCTCTGCCTACAGAGACCCACCAGCTGAGGTCCAACTATGTTCCTGTAC
TGG**CTGGTTT**TGTGTGTCAACTTGACATAGGCTGGAGTTATCAGAGAAAGGAGCTTCAGTTGGGGAAATGCCTCC
ATGAGATCCAGCTGTGGGGCATTTTCTCAGTTGGTGATCAAAGAGGAGGGCCCATTGTGGGTGGTGCCATCCCTG
GGCTGGTAGTCTTGAGTTCTATAAGAGATCAAGCTGAGCAAGCCAGGGGAAGCAAGCCAATAAGAAACATCCCTC
CATGGCCTCTGCATCAGCTCCTGCTTCCTGACCTGTTTGAGTTCCAGTTCTGACTTCCTTTAGTGATGAACAGCAAT
GTGGAAGTGTAAGCTGAATAAACCCTTTCCTCCCCAA

Sequence Position	Motif	K-mer	Z-score	P-value
931	uukruuu	cugguuu	2.198	1.40E-02

>Xaf1

GATGGCTCAGCGGGTAAGAGCACCCGACTGCTCTTCCGAAGGTCCGGAGTTCAAATCCCAGCAACCACATGGTGG
CTCACAACCATCTGTAATGAGATCTGGCTCCCTCTTCTGGACTGTCAGAAGACAGCTACAGTGTACTTACATATAAT
AAATAAATAAATCTTTAAAAAAAAAAAAAAACAGCATCTCATGGACAAGATATCTTCATTTTAAAAAAAAAAAAAAGA
AACTCCAGTGAGGATGGCCAGCTGGATTCCGAAGATTGAAGAGCACCAGTTCCCACTAACTTCCCAGATCCCACTA
GCTTCCCAGTTCCCACTAGCTTCCCAGATCCCACTAGCTTCCCAGATCCCACTAGCTTCCCAGTTCCCACTAGCTTTC
CAGCTTCCTTTCCTAGTGGTCACAAC**TTTGTTTTCTTT**ACCAGTCTCTGTAACTGAATTCCCCCCACCCT**TTAATTT**CT
AACTGTTTCATATGAGTTTACAATGTATTGTAATCATGCTTATCCCCATTGCCTTCCGTTGCCCCAACCTACTCCTAT
GAACCTCTTTTCTTCCCAATAGTTCTCTGCTTTCACATCTTGAGTCCATGTTTGCGGGTGCTGTGTGTGCACGCGCAC
ACACACATGCTAACATGCACATGCTTATCCAGGTCTTATGCCAACAGTAACCACAGCTGATGTGTGCCCGTTTGCA
ACAGCCATGGCCATGCGGAATCCAGAAAAGAGCATCTGATAGTACCCCACCCAGCCCCTAATACTTTGGCTCTTGC
CGGCTTTGGGCCTCATCTTCCACAATGTCCCCTGGGCCTTGGGAGGGATGTGTCATCTTGGGCTCAGGTCAGGGTC
ACTCAACTGTCTCAAAGTGCTCTTGGCACTTTGGGTGACCAAGAAAAGAGACAGGCTTTCATCT**TTGGTCT**TCAGA
AGATGTCTTTTCCTTCTTCTGAGCTACAGATGACCTGGAGAAAAGGCTTTCGCTTCTTGCACTGAACTCGAGCACCT
AGGCTCAACTGCTGGGTTGGTGGCTCAAGAAACCCCTGCTTTATCCTAAGTTTCTCCCTGACCTT**TTTCTTT**GCCTTC
CTCATTGGCTCTTCTGGACAAGACAGGTTAGGAATATGAGGCCTAGAGGGGGAGATGAAGCAGGAGAAAGGAA
GGAAAGAGGGAGGGAGGGGAAAGTGGAAAGAAAGGGAAAGAGAAGGAGAGGGGGAGGGAGGGGAGGGAA
GGGAAAGAATGGGAGGGGGAGGTAGGGAAGGAAGGGGAGGGGCAGGGCGGGGCAGACCAGGGAGAATTAG
AGTTCCCAGAAAGTCTTTATACTGGGAGTTCTTAAAAGACTAATTGTTAGAAGGGCAATCGTCATAGGGAGGTTCA
CATGGTGCAGGGCCATCTTGAACTATCTTAGCCCCTTAAAAAGAAGCCATTTGCCCACATCTGTGTGTGACCTAAC
ATCCTGTGACTCCTAGGTAAAGTTTTGAAACATATGAGTGGATCCCAACTGCTAAGAAAGTCATCACATATGCATC

TGGAGCCTATAGCAAGGTCTCCCGCTTTGCTATAACCGCTCACCTGTTGTACCACCAGAATTTGTAAAGTGACTTTC
TTTGTTTTGTTATTTTAAAACTTCACAGAAATTGTTACCAAGGTCAGAGCACAGATATCTGAAGCAAACTGAATCTG
TATTTCTGGACCACAGTCACTCATTTGGCTCCAAAATAAACTTATCTCTGCATCCTT

Sequence Position	Motif	K-mer	Z-score	P-value
**409**	**uukruuu**	**uuuguuu**	**3.538**	**2.02E-04**
**410**	**uukruuu**	**uuguuuu**	**3.473**	**2.57E-04**
**414**	**uukruuu**	**uuucuuu**	**2.989**	**1.40E-03**
**453**	**uukruuu**	**uuaauuu**	**2.484**	**6.50E-03**
**1064**	**uukruuu**	**uuucuuu**	**2.56**	**5.23E-03**
**1590**	**uukruuu**	**uuucuuu**	**3.462**	**2.68E-04**
**1594**	**uukruuu**	**uuuguuu**	**3.769**	**8.20E-05**
**1595**	**uukruuu**	**uuguuuu**	**3.703**	**1.07E-04**
**1603**	**uukruuu**	**uuauuuu**	**3.44**	**2.91E-04**

>OAS2

GTAATGCAGACCCCCGGAAGCTGTGGAGGTCAGATCTACCCCACTGTGGGTGGAGTTACTAAGTAGGAGTCCATT
CAGCTCTGGAAGACGCTTCTGGAGTGATCTGGCAAAGACTCAGACTGTGTTAGAAAAGGGAGCCTGGTTCAGTCC
TCTCTGGCAGGCTCGCACCTCTATTCTTCCTTCTTGGAATCAAGACATGGGATTATCCTTCCTCCTCCCCCAGGGTCT
CACAGCACAGGCCCTGCTCTGTGTGAGTGACCTCCTTCAGAGACACTTGCCCCATGCAGCTCGATGGGTTCTGGTT
TTGTCTGTATTCTGTGCAGTTATTTTCCTGCCTCCTGCTCTGTTAGTCTCTAGTCAGCAGCTCCAGACTCACCCTGTG
TCACTAAGGTTAAGGCCCTCCCTAGCCCTTCAGCATTGTCAATCCCAACTAGCCCTCGGAGTCTTCCATTGTGCGTC
TTTGCCTGTCTCTTTCCCTGTCCCTGTGGATACAGAGATGTACCATCCATCCAGCAGCTAGCCAACTCCCCTCCCTCC
ACCTCTGCTGTTAAAACCCTTTCTCTTGGGGAAATGTAAACAATATCTACCTCTCTTAATGTCCCAGGACAAACTAA
GCTGCATTTCTCCCTTCCCTGAGAAGCCAAAGCTTCCCTGATTGAGCTTAGCTGCTCACAGGAGAGGGGTTACAGG
CCTTTGAAGCTGCCACACTAGAAGATCTGCACCCAGCTAGATGGGTGCAGATGGCTTCCCTGGGGCTGCATAAAG
AGAACCCCTCCCCTCATCTTTCCTCCTGTATCCTCTAGCCCCTCTCAGAGATCCTGTGCAATCAGGGCAGAATAGCA
TGCAGCTGGTTGAAACCACTTGCTAAATAACTCAGGTGAGGGTCCCATAACCTTCCCAGCCCACCTCCCTTCCAAG
AGTGAAGATAACAGTCAACAAGCCCAGCTGTGATGTTCATTGATAAGCAGGCTCTGGTGGACTCCTAAAGATGGT
GCCAGTGTGGCTCAGTGAATAGCCCTGCATAACATTTTACACACACCAAATGCTGGTTGATATCTCTTGCTGGCTGC
CCAGGGAGCCTTCACCCCAGGGCTTTAACTGCACAGAGACATGAGGTCTAAGCCCTTCGCATCCCCAAGTAAGGCT
GAGCCTTTTTTCTGCCTGTGCTTGCTCTGATGCATTGAGGATCATGCCTGGCCACTGTGCAACTTTTAAGCAGAGCC
GTGCAACATCCCAGGGAGTTGACTTCTATGTAAACACCTTCATCCATTTCTGATGTATGCTTTGAGGTGGCTCAGGC
TGGGCTAGCCCAGCCCAGACAGAAATCCTAGGCATGTGATTAGAGGATCAGAACCCTTCTGGCCCTTCTTCAGGG
GAGAGATGGGGCTGAAGGTGGGGTTCAAATCTCATGCCGAGTGATGGAACCCGACATCCCTAGGTGCTAAGGCC
CCACCAAATTCTCTGGATAAGGAAGTTCCAGGAATCTTTACTGATAAACATCCCAATGTATCAACAAGGTAGACTCT
GACCTCCATGGGACAGAAGATCCTGGGTCAGTCCCCTCCCTGGGGACTCTGCAGTTGGCTGTTCATTTATATGCTT
CATAATAAATGGTTTCTTTGTGT

Sequence Position	Motif	K-mer	Z-score	P-value
**299**	**uukruuu**	**cugguuu**	**3.099**	**9.71E-04**
**324**	**uukruuu**	**uuauuuu**	**2.989**	**1.40E-03**
**1588**	**uukruuu**	**uucauuu**	**2.703**	**3.44E-03**

>Rsad2

GCGCATATTGTTTAGTGCAGAGCTACTGGGATCCCAACAATATCGCCAAACACATCCCACTTATATTTACACAATCC
TGACACATGACCCCATGGCTGGAGCTTACACACATCACTCACTGTGCTGACTCAAGCTGGAGCAGCAGCAGCAGC
AGCAGCAGCAGCAGAATATTCAAAATAAAACACAGTACACCATGAGAATCTCTGGTCTTCTGGAAAAACACAGAG
ACTGAGCAAACAGATGGGCTCTGTCAACCCATTCCTTCAAAGTGTTGTGACCAGGAGAAGGGGGCATCACAGAAA
TACAGCCAAAGATAGAGAGAGACATGTCGGGATGCAGCGATGGACTCACAGATGCTCACTGTTCTTGCCTAACAG
CCAAGGGGAGTATTTGGACATTCTTGCTATCTCCTGCGACAGCTTCGATGAGCAGGTTAATGCTCTGATTGGCCGT
GGTCAAGGAAAAAAGAACCATGTGGAAAACCTTCAAAAGCTGAGGAGGTGGTGCAGGGATTACAAGGTGGCTTT
CAAGATCAACTCTGTCATTAATCGCTTCAACGTGGACGAAGACATGAATGAACACATCAAGGCCCTGAGCCCTGTG
CGCTGGAAGGTTTTCCAGTGCCTCCTAATTGAGGGTGAGAACTCAGGAGAAGATGCCCTGAGGGAAGCAGAAAG
ATTTCTTATAAGCAATGAAGAATTTGAAACATTCTTGGAGCGTCACAAAGAGGTGTCCTGTTTGGTGCCTGAATCT
AACCAGAAGATGAAAGACTCCTACCTTATCCTAGATGAATATATGCGCTTTCTGAACTGTACCGGTGGCCGGAAGG
ACCCTTCCAAGTCTATTCTGGATGTTGGCGTGGAAGAAGCAATAAAGTTCAGTGGATTTGATGAGAAGATGTTTCT
GAAGCGTGGCGGAAAGTATGTGTGGAGTAAAGCTGACCTGAAGCTGGACTGGTGAGGCTGAGATGGGAAGGAA
ACTCCGACCAGCTACAGGGACATTCACGCCCAGCTATCCTTCAACAAGCTACATCTTCTGGCTATCTACGGACTGTT
GTTATTAGAATACAGTCTG**TTTATTTTTCTTT**CAGTTTGACTGAACTAAAAAGGGAGTAAAATTATGCAGCAGATAA
TCGTCCTCCACAGGAGAGAGTTCGGTTCTCAGAGGAGCAAGGTCCAGTGACCCCTAAGAACCAGCAT**TTCATTT**AT
ATTTGAATTTTTGTGTGGATTTTACTGTGAATCCCACATTTCCTTTTTCCTTTTTGTTCCCCTTGAGAAACTGGG**TTAT**
TTTTTGCTAACTATGCCAATTTTGTCAACAGGAAACTTCACAAAACACACCCTCCAATTACTGCTGACTATTCTAATG
CTTTTCTGCTTTGGAATATTGGACTTTTTACTCAAAAGTGTAGAATCAAGGCATATCTCATAGAGCCTTAAGTTAGA
ATCTTACTCTTATGGAAGGAGTTATTTCCTAGAACAGGCTGTTTGTTCATGGTCCCAGCCTCTGATGCTGCTGCTTC
AAGATGGCTGCTTTATACTGCAGCAATTCCTTCAGGGTTCTTGACAAAGCCAAGGTACACTTTACCTCCCAGGGTG
TGGCCATGAGGATGCCACTTCTGCAGCTGCAACTGCCCTGGCATTGAGGCTGCTTGAGTCTCTCATGCTGAAAGGT
TGTCACACTTTTCTGAAAAGCTCCAAGGCCATGTGGCACGTCCAGAAGAGTCGTGGGGGGATAGACAGGAAGGA
GTCTTTCAGGAATATAAAATGTGGCAGCTGGC**TTGGTAT**AAATGTGCCTGTGGTTCCTTAGCATATCTTTCAAGAA
ACACTGAAACTTGACCTAGTCTAGGTCAACATGAGAAAAACAAAAAAACAAAACAAAACAAAACAAAAAAAAAAA
AACAAAAATAACAAAAACAAAACCAAAAACAGTTCCTTCTTTACACCAAAAAGGCAAAAGACAGAATGTGAACTA
GCCAAAATCTTCCTGTGGAAGCATTGCATGAGGTTATTAAGCCTAGACAGTGCCTCACTGCTTGGAAAATAATTTC
CTATCAAAGAGTAAAGCCATGGAAAATTTTGTTCTGTCCAAGAAACTGAACTCCCCTGTAGTTCCACTGTGGCCGC
TAGAGGGCCCCGGGAAGTCAGACCTCATTAAGTATCTCAGAAACTTTGCTTCTGAGTCTATTTTTAGGACTTGGTG
GATT**TTGGTTC**TGTTCCTTC**TTTGTTTTATTTGCTTTTGTTT**GGGG**TTTTTTTT**GGTGCTTATATTAATACTAAAATTT
TTCTAAGGAACTGGAATTTGGGGTATTTACTCACAAAAGATGTTCATACTTTAGTTAGGTCATTATAATTCAACTGA
GACACACTTTGTCTCATGAAAAATATAGGTCAGGGTTTTCTGTGTTTTCTAGTGTGTGTGTGTGTGTGTGTGTGT
GTTTTACCTATTTTTGCTTGAGTATAAATATAACTATGTAGCCCAGGCTGGCCTGGAACTCATGATACCTCTGCCCT
AACCTCACCCTCTCAAGTGCTAGGGATTGCACGTGTGTATTAACCAACTCCTATGTAGATTTTTCTATGTGAAATTT
GAGTCACATTCCCCCTGAAGATT**GTGATTTTTTTTTTT**AGTTAAGTCCTGTGTGCTTAGTTCACCTGGGAAATCCCA
GAGGACATGGCCAGACACCCACTTGATCAAATGAGAGGAAAGCAATCATGAAGCTGAGGTCCAACCTTCTGAATT
GCAGTATATTTTCTCCTGGCTGAGGGAAGCTGCATGTCTCTTTCCCTGATTAAAGTAGAACATGATGAGAATCTGCT
TGGGAAATGAGCCACCATTCTAAATGTACCCCCCCACCCCCCGCCTTGTGCATGCACAGGAGGTGTGTGAGATCTC
TGTGCTTAAAATATTCAAAAATCTCCCTTTCCTAAAGGAAAGAATTACAATATTTTATTATCACAGTAAAGATCAAA
C**TTGGATT**AGATCATTCA**TTGGTTA**CATTTTCAGTGTTAAATCTGGGTGCTCATTTTGTGAAGCAGATGCTCTCTGC
TGGGAGAATAGGGGATGATCTAGTGGACATTTGTTAAGTGTTACAGGCTGATGCCTTGTGTCCCATGACTGATTG
ATGCAGAGTAGTAATGCTGTGAAGGGGGTTTTACTCAAGAACTTCAGAGGCTAGGACATCTCACA**CTGATTT**TACA
GCCTTTGGTGTCTCTGTTATAATGTGTTCAATGATAAAATCATGGGCTGTGTTTTTGTAAGAGGGACGCTTCATGGT

GTTATTTGAAAAAAAAAAAAAAAGGTCCCAGGTTTCGATCTGTTTGGAGTTTGGAGTCTAATGGTTGCATAGATAAA
CAGGCGACCAATCACACCCAGCAGCAGTTAGCTCCTTACCGCAGTAACTCAGCTCATGGACTGCCCTCTTGATGGA
TTGAT**GTTGTTT**CATAAGCTATTATTAGACAAAGAACCATCACGCAGGCCTCATGTGAGTATGTCCAGATGAGGTC
TAATGCTCAGGGATATATGTATATGTATCTCTCTGTGGAATTACACTGTGTTCTGAGGCATCGCTCTAATGTATGGT
AACATGATCTGTGAATTAAAATGTTCTATACGTGG

Sequence Position	Motif	K-mer	Z-score	P-value
1075	uukruuu	uuuauuu	3.319	4.52E-04
1076	uukruuu	uuauuuu	3.176	7.47E-04
1081	uukruuu	uuucuuu	3.176	7.47E-04
1200	uukruuu	uucauuu	2.923	1.73E-03
1284	uukruuu	uuauuuu	2.835	2.29E-03
1778	uukruuu	uugguau	1.846	3.24E-02
2204	uukruuu	uugguuc	3.549	1.93E-04
2220	uukruuu	uuuguuu	4.033	2.75E-05
2221	uukruuu	uuguuuu	3.923	4.37E-05
2225	uukruuu	uuuauuu	3.846	6.00E-05
2230	uukruuu	uugcuuu	3.857	5.74E-05
2235	uukruuu	uuuguuu	4.033	2.75E-05
2246	uukruuu	uuuuuuu	3.637	1.38E-04
2247	uukruuu	uuuuuuu	3.593	1.63E-04
2610	uukruuu	gugauuu	3.198	6.92E-04
2614	uukruuu	uuuuuuu	3.341	4.17E-04
2615	uukruuu	uuuuuuu	3.341	4.17E-04
2616	uukruuu	uuuuuuu	3.341	4.17E-04
2617	uukruuu	uuuuuuu	3.341	4.17E-04
2618	uukruuu	uuuuuuu	3.341	4.17E-04
2970	uukruuu	uuggauu	2.791	2.63E-03
2987	uukruuu	uugguua	2.56	5.23E-03
3431	uukruuu	guuguuu	2.385	8.54E-03

>Ifit1

ATGCAGCTCACCTCTGTGACGTTAATATACTCACAACCAAGTGTTCCAATGCTCCTTC**CTGATTTCTTT**TCCCAGGTC
TGTTTTGTTGTTGTTGTTG**TTCGTTTTCTTT**CTGAAATGCCAAGTAGCAAGGTACATTTTCTCCTGTGACTTCTTTCTG
GTCTTTCTGACCCTGTCTAGCAGGCAATTCCATCCCAAGGATACATACTCACTTAAAGATCACAACTGATTCCTGCT
GTTTTGGACTCCTGTGAGCTGTAGAATCACACAACCTTTTTCCTTATGTCTGTGCCTTTGGAACAGATATCTTCCACC
CTTTGCTAATGTCCTGGACAAAAAAGTCTTTGCCAAAGAAGGTA**TTTTTTTTTT**AATGTCACCGGAAATGCTTATTA
CCATACAATTTCAGAGCAAAACAAATGAGCAAAGAAGAAAGTTTGAAAGCAAAAGAGGTCACACATTACTGGAAA
GATACTAAGAACCAAGAAGACTTTTGGCTTACTAGAGAATTAAAATGAAAACCCTCCTTAGGTAGTAAAGTCTGAA
AATTACATAAATAACTTCTTTCAATATTTATATGCTAAAAGTATTCATTGAAAGTGACCAAAATAGTTAAAGCCAAA
ACCAACAAAAAAAAGAAAGAAAGAAAGAAAGAGAGAAAAGAAAAGAAAAGAAAAGAAAAGAAAAGAA
AAAAAAGAAAAGAAAAGAAAAGAAAAAAAGAAGAAGAAAGAAAAAAAAAGAAAGCGACCCACAAATAACAAAT
TTGGTGTAGTCTATTAATGTTGTGCATTATAAAGGATGCACCTCTATGTTTGAGCAGTTTC**TTGGTTA**TCTTTTATCC

TCAGCTTGATATATCCTGGGACATCTGAAAAGAGCTGCTTGCACCACACTAGCTTGCAGGCATATTGTGGCACTTTT
ATCCTGGATTCCTGACTGTTGTCGTATGACACAGTCAACTGTGAGTGCTTCCATCCCTACGCAGGTAGTCTTGGCCT
ATATATGAAATCTTGCTGTGCATGTGCCAATGAATGAACCAGCAAGCAGAATTACTCCATGATTCCTACCCAGTTTC
CTGCCTTGACTTTTCTGCCCTGAGTTTCCTCCCTGATGGATTATGATCTCTAAGTGTAAGTCAAATAAACATATTCCC
CTC

> Fabp4

GCCAAAGGAAGAGGCCTGGATGGAAATTTGCATCAAACACTACAATAGTCAGTCGGATTTATTGTTTTTTTTTAAA
GATATGATTTTCCACTAATAAGCAAGCAATTAATTTTTTCTGAAGATGCATTTTATTGGATATGGTTATGTTGATTA
AATAAAACCTTTTTAGACTTAGAAAGTTGTGTAATGATACTTTTATTGTGTGTCTAATTTTGTACTTGTAATTGTGAA
GATAATAGAACAAATTGCATTACTTTTATTTTGGTTCTGGTACAAATAATATGATCTTTCTTAATAATGCATGATAA
AAAAGCCAATGTCCATGAACTTACCAAGTCTGCATACTAAGGTCCGCTGTGGCATACAAGACCTTGGGTAATCCTA
GACACAGTGAAATTATTCATCTCCATATGATTAAAGCATGAAGCTTTG

Sequence Position	Motif	K-mer	Z-score	P-value
61	uukruuu	auuguuu	3.44	2.91E-04
62	uukruuu	uuguuuu	3.582	1.70E-04
65	uukruuu	uuuuuuu	3.462	2.68E-04
66	uukruuu	uuuuuuu	3.462	2.68E-04
67	uukruuu	uuuuuuu	3.462	2.68E-04
80	uukruuu	augauuu	3.451	2.79E-04
106	uukruuu	uuaauuu	2.703	3.44E-03
256	uukruuu	uuuauuu	3.341	4.17E-04
257	uukruuu	uuauuuu	3.198	6.92E-04
262	uukruuu	uugguuc	3.242	5.93E-04

>Hdac9

AGTGCCAAGTCTCCCCCGATATTTCCTGTGTGTGACATCATTGTGTATCCCCCCACACACAACCCCAGCACCCTCAG
ACATGTCTGCTGCCTGGGTGGCACAGTTTGATGGAACATAAACACTGGGCACAAAATTCTGAACAGCAGCTTCCCT
GGTTCTCTGGATGCACTTGAAAGGGCATTACAGATTCCCGAAATGTAACCACTGTGTATCTAAAGTTACAGTAACC
AGGATTGGAAGAAACTGTTTCCAGCATGCTTTTCTGCTGATGACCCACTCCCAGACACAAAGTGTGAGCTAGAAAC
CTTCAATACAGCATTCGATATTGTTCATTTCAGAAGCTATGACAGCCAGTGAAATTTGGGGCAAATCCTGAGACGT
ACCCATTCCTGACATTCTGACCAGTGTCTTATTGTGTAGTTTACTGTCAAGTAGACTTAAATTGTTTACAGGACTTCT
TTTCAGCTGTGAGTCGCTGGGAATTACAGACAGATTGCTGTGGGTTGTAGGGAGTTTTTTTCTCTTTGTTATACTTT
TGTTTTGTTTTGAACTTTTTGCCTTTGTTCTAATACACTTCAATCCCTCCTGGCAAGAAGGGACCTGGGATAAGACG
TTTTTCCTTTTTCCCTTTTTTTTTTGGGGGGGGGGTGCGGTGGAGCCATGGTTTCCCTGTGTAGCCCTGGCTGTTCT
GGAACTCATGCTGTAGACCAGGCTGGCTCTGAACTCAGAGATCCCCCTGCCTCTGTTCTAGGATCAAAGGAATGCA
CCACCAGCACCCAGCAACAATGAGGCTTTTTTTTTCCTCGATGGAAAAAATATTACAGTGTATCTGACAGTTCAAAA
GGAGTTTGCACACTACGCGGGCAATGTGGCAGTATTTTGTGTCTACTTTTACTGGAAATTTACATCTGTCTCAGGTT
CTCACTCACAGTATATGAGCTTGCAGTCTTGACAGGTAATGAGGTCGCACCACCCCCCATGCAGCGCCACCTTGAG
GACAGTGGCTCTGGAAGTGAATCTCTTGGGAGCCATGCAGCCCTTGCACAGCTCAGAACTTCTCAAGGGTGGTAG
CTAGCTCCAGCTCTATCTGGCGCTCATTCTCTCTTCTTTCCTTCTCAGCCTTTCTTGTCACCCTTTTAGAAAACTCACC
CCAACTTCCCCATTACTCAGGCGATTTGAGTATTTTATTTATTTTCTTTACTTTTCGGGTGAACCCACTCGTGTTATCT

TCTGCTGTGGAACTACTTTAGTGGGT**ATAGTTT**AAGTGATGCCACTGAATTCCCAGGTCTTAGAAACATGTCAATG
AAGATGACAACGGTCTGAAGTCCTTTTGAATGGGGCTAAGCTAAGAATTGTGCCTTCACATGAAAACTAACACAG
AACTTAACCTTTGCACGGGTAGTATTTCCAACAAAGGTTCAAAATAAAGGACATTTAATTCTGAGATTAAAAACAG
CTTACTAAGTAAAATATAAACAAGTTAGCCCCTGCCTCTGAAAATTGTATTTCAGTGTATAACTCAAAACACCTAAA
GACAGTGAGTAGAATACCTTGCATATTTAATATCTGACAGTGCTTTTGAACA**TTGATTT**TTAATGTATTTTTCTTAAC
TCAAAGGATATATTAAAGCCATAAGTGAAGATTGTCATGCTTTTATTCCGAAATCTGAAAGAAACCTTAATTAAAAC
AAGGTTTTAGGGAAGGCTAAGATATGAAAGACATGGAACAATGTGGTTTGAGTTAGGGAGGACTCCAACCTGTA
AATCAAAGATGAAAGATCTCACTCACGTAGAGTCACATAACTCCCCTTTGTTATACAGTCAGGCCACATTTTTCATG
CAT**TTGGTTTTTTT**AGGATTACCATT**TTAATTT**GTAAGACTTTTATTACAGATACAAAAATGGC**TTGGTTCTTGATTT**
AACATCTTAGAAATCTGAGATACCCTTTGAAATAGGAAATCTGAAATGGAATGTAACTTAGTATTAGGTAAAAATT
GCTTACATTGCATAAGAATTAAGTCAATCTAGATGCATAATTGTAATAAATTTCTTGTAAATTCCATTTTCTTGATAT
TTTAGAGAAATTCAATTATATTTGCTCTCTCTCTCTCTCTCTCTCTCTCTCTCTCTCATGTGTGTGTGTTTATGTGTGTG
TGTGTGTAATTAGCGAAGTTACTTTAAAAATACCAAAGGCTTACAAAAGACCTTTTCCTTTTAAAGGGGGAAACGT
TATCATACTTCGGAAGGTGTACACTTGTGAAGCTTTAAAATCAGCTACAGAACCACAGGTCCCCATGCTTGCTCTTC
AGTCTTTGCAAATACATTTTAGAAGTTGGGTAATGAAGCAAAACAGCAGGACTATACTACTCTACTGATGCCCCAG
CAACGGCATGGCCTCAGAGGCTGACATGTCACTCGGACAAAGCCAAGTCACTGGGGCATCTTCGTGGTAGTTTAG
ATTTAAAAAATACATATGAAAGCATTAATCGCACACTTCCTTCCCACCATGTGACAACAGCCTGACTGACTGCAATG
CAAAGTATCAGAACCCTGGGAGAGGAACAGATATGGTGCCTTCAGTTCTCGCTATGACCCTCTGGGC**ATAGTTTCA**
TTTGTTTAACTAAAATCACAGAGGCCTTGAAAATAAGGCATCTCCTCCCACAAAAACTTCAAATATTATGGGACTG
GTCTCTCCTAATAGTTCAGA**CTTGTTTTATTT**CTAGAGGCCAATCACAAAGCTTTCTGAGTTCACTGGGTATCTTTAC
AGACTCCATATGGGGAGTGAATATGAGTGACCCCGAGAGTCTCCTGGAAGAAAAGCCTTCTCCACTTAGCATGTTT
AAGCTTTTATAAATTTTATTCCATCACTTACAATTAAGGCAATTTATGTAA**TTGGTTT**TTGTTATTATTTCCCATTTAA
GACTTTCCTCTATTTGTCTCAGTGGAAGC**ATGTTTT**AAAATCTAATAGTCCAAAAGAAACCTGAAAGTGGATCATTC
TGAACTTAAAAACAGCCGTTCTGTCAAAGTGGCACACTGTCTGTTCTAGTCTTAACATTCTTTTTCTGCCTTTGGAAG
TGAAATAATGTGCTCACTACGAGAGACAGAGAGAGAGAGAGAGAGAGAGAGAGCGAGAGAGAGAGAGAGCGAGC
GAGCAAGAGAGAGAGAGAGAGCGAGAGAGAGAGCGAGAGAGAGAGAAATGACATAACTGCTTTACCACTTCCATA
AAGAGAGCATTTTTACCACCAAAACAAAACAAAACACCTAGAAAACCAGATTAAGGCTCAGAACAGAGGCATTCC
GTTGCCTTCGACGCTGCTCATTACTTGTGGTGGTGGTCTCACTCAGCATTAGCCCTTCTCTCTTCCCCAAGGACACT
GCTATATT**TTGGTCT**CTAGTAGAAATCTAAACTATTTACTGCCATGTAGCAGAACTTATATTGAGGATTTCATCAAC
TTCCTTAACACCATCACTAAAATATTTAAATGTGTATGATGTGGCTCTGTTAGTCAGATGTATTTCCATTGGAAATCA
GAGACTTTGTCTCCCAGAGCCAACATC**TTGGTTC**TGCTCTATTGAATATTTCATGAGTCACTGAGTGACTGTGTGTG
ACCAGAGGATTGTGTGTGTGTGTGTGTGTGTATGTATATGTATATATACACATCTCCACATGTGCTAAATAAGGTC
AGTTGCTTTCCAGAATACTGGGACCAAGGGCCATTACATTTACTGCTGTACACCCTGCTCTTTGATGGAGATAGCAT
TTTGTAACAGATGACAAGGCTGTTCCAGCAATTGAGGCCTTTATTAACCTAACATTTAATAAAAATGAAAAAAAAC
AAAGGATTTAAAAGCTTTATAACCTGAAGAGAAATTTTACTTCTTTGTGTATGCAGAGATTTTTTAAAGAGAGAGA
AAGAGGGGGGGGGAGAGAGAGAAGTCAGTTATCTCTTTTTAAATTGTAAGGTTCTCAAACTCTTTCTGGATGAGT
GGCCTCAGTGCAGACCTTCACTTCAGTGCAGAAGCTGGGTGAGATGAGACTGTCTTTCCAGTGGTAACTGGGCTT
AGCAAGTGTACCATCCAAAATGACAGACCTTTGTGTAGGATATGTGCCAGGCAGTCTGTGTCATTCAAAACTAATA
ACCTGACATTTCAAATCAGTTGGACTCTTCAGGCAAATATGCAACAAAAGCGTTTA**TTATTTT**GCTGATTAAATGCT
ACACCAGGATTGCTGGAGTGAATGCTGATAACCTGGAGTTTCTGGTTCCTGTAATGTGAATGTGTCTTTTGTATCAT
GGTCTTGTATCTGTTAACGTAAGAAGATCAATTGATTCTCCTCTGTGAACAA**GTTGTTTT**GCC**TTTTTTTTTT**ACTTT
CATCAAAATCTGTCATCCAGAACAGAAAAGTATGCATTGCCAAACATGAAGTTGTTCTGCCAGTGGTGTGCGCCCA
GATGTGCAAGCTAGTGCCTAATAAAGAATGAAAAACTAATAACAGCACCATTGTTGCACACGCCTTTGGAATGTTC
ACCTCCTTTCCGCTTCCCTCCTGTTTTGTTGTGTTGTGTTGTGT**TTTGTTTT**GTTTGTATTTGGAAACACGGTATCATG

CTCCAAAGGGTGGCTGATGTTGTTAGTGTCACCACGCCTTCAGAAGAACAATGACGCCCATTTTGTGTTTTCTA**CTG**
ATTTCCTCTAGTCCCATGTGAATAAAAGCTATTTGAAATAA

Sequence Position	Motif	K-mer	Z-score	P-value
442	uukruuu	auuguuu	2.945	1.61E-03
514	uukruuu	uuuuuuu	3.516	2.19E-04
535	uukruuu	uuuguuu	4.022	2.89E-05
536	uukruuu	uuguuuu	3.912	4.58E-05
540	uukruuu	uuuguuu	4	3.17E-05
541	uukruuu	uuguuuu	3.89	5.01E-05
629	uukruuu	uuuuuuu	3.264	5.49E-04
630	uukruuu	uuuuuuu	3.264	5.49E-04
631	uukruuu	uuuuuuu	3.264	5.49E-04
632	uukruuu	uuuuuuu	3.264	5.49E-04
793	uukruuu	uuuuuuu	3.022	1.26E-03
794	uukruuu	uuuuuuu	3.022	1.26E-03
795	uukruuu	uuuuuuu	3.022	1.26E-03
1184	uukruuu	uuuauuu	3.495	2.37E-04
1188	uukruuu	uuuauuu	3.495	2.37E-04
1189	uukruuu	uuauuuu	3.297	4.89E-04
1193	uukruuu	uuucuuu	3.33	4.34E-04
1586	uukruuu	uugauuu	3.209	6.66E-04
1843	uukruuu	uugguuu	3.901	4.79E-05
1847	uukruuu	uuuuuuu	3.571	1.78E-04
1866	uukruuu	uuaauuu	3.626	1.44E-04
1904	uukruuu	uugguuc	3.055	1.13E-03
1911	uukruuu	uugauuu	2.67	3.79E-03
2600	uukruuu	auaguuu	2.89	1.93E-03
2605	uukruuu	uucauuu	2.945	1.61E-03
2609	uukruuu	uuuguuu	3.165	7.75E-04
2705	uukruuu	cuuguuu	3.011	1.30E-03
2706	uukruuu	uuguuuu	3.286	5.08E-04
2710	uukruuu	uuuauuu	3.044	1.17E-03
2890	uukruuu	uugguuu	3.637	1.38E-04
2947	uukruuu	auguuuu	1.923	2.72E-02
3378	uukruuu	uuggucu	1.879	3.01E-02
4189	uukruuu	uuauuuu	2.044	2.05E-02
4339	uukruuu	guuguuu	3.341	4.17E-04
4340	uukruuu	uuguuuu	3.626	1.44E-04
4351	uukruuu	uuaauuu	3.527	2.10E-04
4355	uukruuu	uuuuuuu	3.495	2.37E-04
4356	uukruuu	uuuuuuu	3.495	2.37E-04
4357	uukruuu	uuuuuuu	3.495	2.37E-04
4358	uukruuu	uuuuuuu	3.495	2.37E-04
4359	uukruuu	uuuuuuu	3.495	2.37E-04
4360	uukruuu	uuuuuuu	3.495	2.37E-04
4361	uukruuu	uuuuuuu	3.495	2.37E-04

**4569**	**uukruuu**	**uuuguuu**	**3.934**	**4.18E-05**
**4570**	**uukruuu**	**uuguuuu**	**3.824**	**6.57E-05**
**4574**	**uukruuu**	**uuuguuu**	**3.934**	**4.18E-05**
**4678**	**uukruuu**	**cugauuu**	**2.67**	**3.79E-03**

>Dmxl2

TCTGAGAGCTGGGGGTTATTTTATATACCTCTCAGTTAAAAGGTGTAATGTAGGAATTAGGCAGTTCTGTTTTCCAC
GACAGTCAACACCTTGAAATCAGCAGAGAGAGTCTCTGTGGAATTTGACTCTGATGCAAGCTGGGTTTTACTCTGA
GTGTTGCCTACCCAGGTTGGTAGAGTGCATGTTCCTGTCATTATACTGATGTATTAAAACATAAAAACCCTACCTTG
TATCTTATGAAGGGAAGCTATCCTTTACGCCATGTAACAACCCTGATTCAGAAACACTTGAGGTTAGCTAACAGTA
AGTTACAGTAATGAGCAGGGCCTTTATACCAAATGGAGGAAGAAAACATTGCTGACTCACGTTTTCCTTCCTGTTC
TCATCATGGATGGAGCATGCCGCTGTCTCTTGTTGAATGAAGGATAGCCCCGGGGTGTGACGCTCTAGAGCACCA
GAAACCCTTCCTAACTGTGAGCAGGCCTGGCCACTCTGATTCTCCCTGCTGAGTTTGTTCTCCTGTTAGTGTTTATAC
TGAGCTAGTACTGTAACTTGCAAGTGAGTGCAAATTTAAATGCAATGTTTTCCTCACAATTTGCACATTGACATTTT
TTGGACTGCTAGTTTTTCTATTAAAGTATTTGCCTTTCTGTCAGGAATGTAATATGTTAGACATGGCGTATGTGTAG
TTAAGCAACACATACCTTCTTAGTCTAGTTTAGTACTTTTTCTTTTCATGTATTTAAGGTTAATAAGGCACATTTAAA
GCTATGTTGAAACTACACCAATAGAACATTTCCTATCAATTAAATGAATGTTAGGCTTTTTGTGGCCAGTTGATGAG
ATTGGTGATATTATTTATTGCCACAGCCTATTGTATAAACTATGCAGCGTTAAATATTATTTGCTTGTAAAATATTAG
CCAGTGTCATATTTTGATGTATTTCCTTCGTTATGACCAAAAATATGTTGCAATATTGAAACCAGTGTCTGTGTGTGT
TTAAGTGTTTACCAGCAAATTGTCTCATCATGTAATGAGAATGTTTAATGCCCATGTGGAAAATAGTAAACATGAC
GGTATAGAGTACCTTTCTCTGAAGGCAATGTGTAATCATGCTGTGAAATACATATGTAAAAGAAATGTTATTTGTT
AGAGTTTTATTTTAGTTTGGCCTATTTAAGTAGACTGGTCTGGCATTATTTGACACTGTTTTTAAGAGTTAATTTTAA
TAAAATGATGAACTATTAGAGAAACTTTAA

Sequence Position	Motif	K-mer	Z-score	P-value
728	uukruuu	uuucuuu	2.934	1.67E-03
1158	uukruuu	uuuauuu	3.484	2.47E-04
1159	uukruuu	uuauuuu	3.286	5.08E-04
1164	uukruuu	uuaguuu	3.571	1.78E-04
1221	uukruuu	uuaauuu	2.253	1.21E-02

Up-regulated genes, Consensus motif (uukruuu)

>Mmp9

ACTAGGGCTCCTTCTTTGCTTCAACCGTGCAGTGCAAGTCTCTAGAGACCACCACCACCACCACACACAAACCC
CATCCGAGGGAAAGGTGCTAGCTGGCCAGGTACAGACTGGTGATCTCTTCTAGAGACTGGGAAGGAGTGGAGGC
AGGCAGGGCTCTCTCTGCCCACCGTCCTTTCTTGTTGGACTGTTTCTAATAAACACGGATCCCCAACCTTTTCCAGCT
ACTTTAGTCAATCAGCTTATCTGTAGTTGCAGATGCATCCGAGCAAGAAGACAACTTTGTAGGGTGGATTCTGACC
TTTTATTTTTGTGTGGCGTCTGAGAATTGAATCAGCTGGCTTTTGTGACAGGCACTTCACCGGCTAAACCACCTCTC
CCGACTCCAGCCCTTTTATTTATTATGTATGAGGTTATGTTCACATGCATGTATTTAACCCACAGAATGCTTACTGTG
TGTCGGGCGCGGCTCCAACCGCTGCATAAATATTAAGGTATTCAGTTGCCCCTACTGGAAGGTATTATGTAACTAT
TTCTCTCTTACATTGGAGAACACCACCGAGCTATCCACTCATCAAACATTTATTGAGAGCATCCCTAGGGAGCCAG
GCTCTCTACTGGGCGTTAGGGACAGAAATGTTGGTTCTTCCTTCAAGGATTGCTCAGAGATTCTCCGTGTCCTGTAA
ATCTGCTGAAACCAGACCCCAGACTCCTCTCTCTCCCGAGAGTCCAACTCACTCACTGTGGTTGCTGGCAGCTGCA
GCATGCGTATACAGCATGTGTGCTAGAGAGGTAGAGGGGGTCTGTGCGTTATGGTTCAGGTCAGACTGTGTCCTC
CAGGTGAGATGACCCCTCAGCTGGAACTGATCCAGGAAGGATAACCAAGTGTCTTCCTGGCAGTCTTTTTTAAATA
AATGAATAAATGAATATTTACTTATTTA

Sequence Position	Motif	K-mer	Z-score	P-value
307	uukruuu	uuuauuu	3.011	1.30E-03
308	uukruuu	uuauuuu	2.879	1.99E-03
397	uukruuu	uuuauuu	2.516	5.93E-03

>Postn

AAACCCAGAGGCCAGACCACAGAGTTTATATAATCCTAAATCAACGATCTGATTTTAAGGGAAATTGTAAGAGCCA
CCACACTGACTTCAGAATCTGAAATGACAACCAACAGAAGCCAATCTTCAAGCAAGTCCAAACACAGAGTTCATGT
CTTTGTTTCTGCATGAGAAATATAAGAAAATGATAGCTAGTCTCCTGTGGGGTAGGAACTGAGGAAATATAGGAC
CATGCAGGGATTTTATCTCAATGAGAAAACTTCTGATTAAAGTAGAATCCACCAAAGAACATCATTGTGACTGGGT
CCATACAGCTAAGTCTTTGCACAGTAAAAACCTTCCGCCTCAGGAAGAGGCTGGAAAAACCCAAAGCACACAGTT
ACCTTTCCAGGGGAGGCTAAGGTATCAAAAGGGGTGTTCAGTTATACAACATGCAAACAAACCTACCAAATTACG
AACAGTGGTGTTACATATTTCTCATGCAATGTGGGTTTCCTGCTAAATTTTGTTATTTTTACACTTGATTTATATCCT
CGAGATGATTGTCATAAGCTTCTTGCAATACAAATGTTTTCTCTCAAACATTTCAATAAAACCATTCTTCAGGTATAA
AGAGAATTACTGCAGAGTTGGTAATTCAGAAAACTCAAGGTTTAAGTTAAAAGTGAGTTTAGACTTTGGAATAGG
ACTTCATACCTTTTTTTATTGTTAACAAGTACTCAATAAAGGAAACTGAAT

Sequence Position	Motif	K-mer	Z-score	P-value
485	uukruuu	uggguuu	2.967	1.50E-03
506	uukruuu	uuauuuu	3.319	4.52E-04
518	uukruuu	uugauuu	3.495	2.37E-04
695	uukruuu	uuuuuuu	2.692	3.55E-03

>Pcolce2

CCTGAGCTCTGTCACATAACCTGAACTTACTCATCACCGCTGAACGATCTGTCCACGTTAGGAAAAAAAAAAAAAA
AAAACCACGCTATTTAATGCTGAGCGTTCTGAGAGATGGAGCTGAGTAACTCCTCACGCGATGGATGTGTGAGCC
ACGCCCCCAGTTCACTGGTGGAAGGTCTGCGCCTGCGCTGTGATCTCCAACATCTGCGAACTCACCCACCCAGTGC
CTGATGGGGAAGAGCAGCGAGCTCCGACATCTGGAAGCTTGAACATACATCTCTGTAGGAGGATATTCTAGAGTC
GAGTTGTATGAAGATATACAAAGGATTTTATAAAGTGCAATATTTATAATGATATTTGGTTACCTTCAAGCATTTGC
TCTGAGGTGTCACATTCTTTTTTTCTCTTTCATTTTTTTAAAAGTCAATGCTTAATAAAATATTTTAAATGATTATAAG
GCAGCCAGATAACCTTGTTCTCCCAGAAAATACAACTGTGGAGCCAGTCCCGTGAAACGGTGCTCGAGGTGGTAT
TTGTACATAAAGTTGTCGCTGTATCAGCTTCAAGTTGTCACTAAGAGTTCCAGGGTTAAGACTGATGCAGTGACGT
CAGTGGACTGTAGTCTCTGTCTGCAAGAACAGAAAGGTGTCCCTCTGGCATCCAGTCCAGACTCCTTCATTTTGTCT
CTGAACTGCAAGCTCAGGTATATACAAGGGAATTCCCCAGAGTACACTTCTCAGCAAAGGCAGTGCAATCTTCTAA
GAGTCCTAATTTCAAACCCAAGGTCACAATGTCGCTAGAGAAGGCTCTCGAAGCCCCAGGAGGAAGGCACCTTCC
CACTTGAAGCATTTAGTACTAAGGACTGATGCTTAGGCTTGCATTCGCTAGACTAAGCAATAAAGTAATCTAAAAA
TTCAGCAGTGAGCTCCAAGTACTCTTCGCTGTTTGTACCCATTGTACTGAATCAGACTGTATCAGTTGAGATGGACT
GCTCGAGCGGCAGCTAGACCTGCAATGGGTATGACAGTGTCTGCCTCCTAGGAGATTGCTGGCCAGGGGCTGTGA
AAGGTGCCTTGTCTCCATCGAAAGATGGTACGAGGAACCCTAGATAGACAACGGCACGAGTCACAAGGTCTCTTA
TGAGAGGCATGAAGTTGTAGAACTAAGGGGAACGCTTATTTTGGTTGGAAATGGCAATTCCCAGTTCTTGAGCTG
ACAAACACTTACGTGACAGCAAGATATTAAGGTAAGTTGAACAGCTGTCCTGCCAGCTGGTCAGGTGTACGAGCA
ATTAACAGGAGTCAGTTGGAAGGGACAGAAAAGGTTCAAACTCCTCCTGGGATGCCCATCAGTCCTCGGACCTCA
CCAGCTAAAGGAACGGATGGGTTAGAGCATCCTGCACCAGCTGGGCGGTGGCTTCTCTGGGCAGTGACACCCTGT
AGACAGTCAGACCTGTTAATGGAATCCTGTCAAGAATGCACTGTAGGGACGTCGGAATGAGCACAGGCTGTTGTC
TCTCAGTGGAAACATCTTACTGATGAATTATTATGGACAGCTTGTTTGTGTAGCTTCATGGTCACAGTGTCTCCTGT
GTTACCAGTGGGGTCTGGAGTCACCCTGCAGGAAAAACCAAGAACAGTAATCCAAGCTATCCGTTCCATTGACAG
CTCTGGCACAAGACAACGGCTAAGGGGACTGTGGTTAATAATTCATGACACTGGCTCATCTTTCAACTCAGTTGTT
TAAAACAGGTAGTCCAGCCAGGTGGTAAGACTACTCTCCCGGTCCTACAAGGCTCTAACGTTGGTTATAAAATTGC
TTTATTTAGACAGAATTCCAGTTCCTTGGTTATAAACAGAGGGAGTTGCAGTAGGCACCTGTATCCACTGACCAAG
CCTATATGTAGGATAATTTTAAAAAACTTTCCCACTTAATGTACCATGAAGCAATTTTTAAAGAACTACATCATATG
AATACCTTTCTCCAGCAATAGTGAATAATTAAAGATCAGTGCCAAAAAGGAAAACCAAAACAATAAAATCTTTTTCT
AGAAATTAAGAAATATGCTGTTAAATAATTAACTTTAAAAATCATAACATAAAAGTATTAAAACTAGACACTTTAAA
TTTTATAATTTCATAAATGATACAAACTAAGAAAAAAACCAATAAACTTTTAAAAGGAGAGAGAGAGCTTTGATCC
CAGCATGCAGGAGATGAAGACAGGCAGATCTCTGCCAGTTGCAAACAACCCTGAAAGGTCGGGAGGATTGCATG
GAGGACTGCCATTAAATTAGCTGCCCCCCAAACTTTAGGACCTGAGTTTGGCTCCCCAGCACCCAGAAAACAACTA
TGTGCAGCAGCACTATAATCCCAGTACCGAAAATGCAGTACTTCTAACTAGGGAGACTGGGGAAACTGAGTCATG
CAGGCCATGGAGCTGGCTGGAAGTCCACTGAATTAGCAAGCTCCAGGTTCAGTTAAAGACTCAAAAAAAAAAAAA
AATGCAGTGGAGAATAAGTGAGGAAGACACTCATCGTGGATTTCTGGCCTCTACACACATATACACATATGTATGA
ATACATGTAAATGCATACATAACACACATGCAGGGGGAGAAATACATTTAGAACAGAAATTGAGTCAAAAAGAAA
TTACAAATAACAGATGAAGTTAACAAAGCTAAAAGTTGCTTCTTTGAAGTGACTAATATAATTCAGAAAACTCTGC
AAGATTAAACATTAAGAAGTAGTCAAAAAAACCAAAATAACAAAAAAAAGAGTAGGGGGCTGGAGAGATGGCTC
AGGGTTTAAAAGCACTGACTGCTCTTCCAGATGAACCCCAGTTCAATTCCCATTCGTGGTGACTCACAAGCATTTGT
AACTCCAGTTCCAAGGGATCTGACACCCTCTTCTGGTCTCTCTGGGCATTGCATGCATATGATGCACAGATGTAGTT
AAAACACATGTAAAATAAAGATAAATAAACTATTTTTAAAGAT

Sequence Position	Motif	K-mer	Z-score	P-value
358	uukruuu	uugguua	2.11	1.74E-02
397	uukruuu	uuuuuuu	3.264	5.49E-04
408	uukruuu	uucauuu	3.209	6.66E-04
412	uukruuu	uuuuuuu	3.253	5.71E-04
1176	uukruuu	uuauuuu	2.648	4.05E-03

1181	uukruuu	uugguug	2.681	3.67E-03
1556	uukruuu	cuuguuu	2.352	9.34E-03
1804	uukruuu	uugguua	2.879	1.99E-03
1816	uukruuu	uugcuuu	3.033	1.21E-03
1820	uukruuu	uuuauuu	3.308	4.70E-04

>Col11a1

GATTAGGACGAAGACCTTATGAAACCAACAGGAAAAAAAATACCTTGGTGCCACCAACCATTTTACGCCACATGCA
AGTTTTGAATAAGGACGGTATAGAAAACGTGCTTAGGAATCGCTGTTGCCTACTGGAGGCACAGACAGAGGAAA
GAGGTTTTGGTAGTTATGACGATGTAAGGTAGTGTGGCGGAGATGGCAATGGGGCTAATTCTTGATTCTCAACCC
TCATCTCTCCTTTTCCTAT**TTGGATT**TCGTTGGTGCTGTAGAAAACAAAAAGAGAGAAAAATATATATTCATTTAAA
AAATGGTGCTCATTCCCATCCATCAAAGATAGAGTAAAATTATGTTTAATAAAGTGTAA**TTATTTT**ATGTACAGTTC
TACACTGTCATCCCTGTGTCCATTTCCAAAACTTGCACGTGACTCTCAATCCCACCCAGCTCAGATTTCATGACAATT
GTGGACTGTGACGGCAATAAATATTTGTGATACAAAACTCAAGTTGTATTTCTGCTGACTCTAATTGCCTTTCCTTG
ATTAATAATAAAATGCCTTTGTATATGTTGATGTTGAAGACTTCAGTTATTTGATGTCACTGACAAATTTCACGGTG
GTCAAATCTAGACTTTCAGGATCACACACCAATCGTCCCTTCTCTGCTAACCGTAACTTGTTGCGTTTCAGCCAGAA
GTATCATGCATTTTAATAATTAATTCAGTGCTATACCTCGAATATTTTCTTCTCAGAATCCAGATTTCACCAAATACTT
GTATATATGGGGAACAAGAAAAGTTATATTTTTGGACAGGAAAGTATGTATGAGAAACTGCTTTAACATCCACCCA
GAAAATAACTTTATGTACAATTATTTCTCTACGTAATCTTCTTCATTGTCCTCAGAGTGCTTCAGTAATGCCAACTAA
TACTAAAGATGGAAAATAAGCAATTATTTATAAATTTGTGCAATGTTAGGTTAAAGCAATGAACTGTTAAAGTGGC
ATAGTAATAATAGTCGCAAATA**TTATTTT**ACTTGCCCAACAAATACAGTTCCTTTGTTTTAAAATAA**TTATTTT**CAAC
ATTTGACCAACCTAGTAGCTAAAAAATACATAGGGTGAGCTGTTTCAAAAGTCATATTTGCTCAAGACAATCAACC
CTTCTC**TTTTTTT**CCACTTAGGTAT**TTTCTTT**TACATACCTTGAACTAGCAATACAATTTTAAAATCACCAACTGAATT
TTGTATCTATTTTAAGTAATATATGTAAGACTTGAAAATAA**ATGTTTT**ATTATAATTTCTTATATGAAGTGTTAAATT
AATTGATACCAGTTTCCACTGGAACACTTATCAGCTGATAATTTATCTCAAAGAATATAATCTGTAATCTTGACATTT
AAAAATGAACATTTGTAATTCAGAA**TTTCTTT**ATTTCTGAAACCAAGTTTGTAAATGTCCTTTGGAGGAAGGAGATA
TGAATTTTATCAATAAATCAAGTCTTGTCTACCTGGATTGGTCAT**TTTCTTT**CTTAAACAGGTTGCAATTCACTCTTG
TGTTCAAAAGTACTTTTGATTGTCATACTTGACCATCCAGAGAAGAATTCTGTAACCCTTGCATGTTAACATTCAAA
GATAAGTTCTCAAATATACAGCATAGTAAATAATGGCATACAGTATTTCCAATAAAATGATAATTTATATTTAATGT
CACAAA**ATGGTTT**TTAGAGGTAAAAGGTTTTACAGGTAAAAGTCTCATACTTTTGACTTTGGAACTAAAAATAATTT
CCAAGCAAATTATTAAGTTTTTAAATCACACGCGAAATAATA**TTAGTTT**ACAAAGTTTTCATATATG**TTAATTT**ATCT
GCATTAATTAAAGACTTTGTTGTATATTAACTTATTCCAAA

Sequence Position	Motif	K-mer	Z-score	P-value
245	uukruuu	uuggauu	2.44	7.34E-03
362	uukruuu	uuauuuu	2.681	3.67E-03
1020	uukruuu	uuauuuu	2.044	2.05E-02
1050	uukruuu	uuuguuu	3.56	1.85E-04
1051	uukruuu	uuguuuu	3.495	2.37E-04
1065	uukruuu	uuauuuu	3.22	6.41E-04
1158	uukruuu	uuuuuuu	3.077	1.05E-03
1177	uukruuu	uuucuuu	3.044	1.17E-03
1272	uukruuu	auguuuu	2.33	9.90E-03
1509	uukruuu	uuucuuu	2.286	1.11E-02
1702	uukruuu	augguuu	3.088	1.01E-03
1815	uukruuu	uuaguuu	1.769	3.84E-02

1840	uukruuu	uuaauuu	2.835	2.29E-03

>Alpl

GGGTGCAGGTCCCACAAGCCCGCAATGGACAGCCAGCTCCCCTCCTTTTGTGGCCCACCACCGGGCAGCCCACACT
CAAGGGAGAGGTCCAGGCAACTTCCAGCAGGAACAGAAGTTCGCTATCTGCCTTGCCTGTATCTGGAATCCTCCAT
GGGCCAGATTCCTGGCTCTGCCTTTATTCCCTAGTTATTGCCCTTTGGCCAGCAGGTTTCTCTCTTGGGCAGGCAAG
ACACAGACTGCACAGATTCCCAAAGCACCTTATTTTTCTACCAAATATATTCTCCAGACCCTGCAACCTCCATGGAA
CATTCCAGATCTGACCTTCTCTCCTCCATCCCTTCCCTTCCCTCTGGAACACTGGGCCCCATAGTCACGGCCAGTCCT
CAAGCCCAACCCTCCCTGGGGGGAAGACCAGGTCTGCTCAGGATGAGACTCCCAGGAAGCCACCTCCGGGGTTG
GCTGTCTACCCAGGGTTGCCAAGCTGGGAAGAACACTCCAGCCGGACAGGACACACACACACACTCCCCACCCAA
TTGCAGAGACTCGCCAACCCTTCACTGAAGTGGCTCTCCTGTTTGGAATAGCGGGGTGGGGTGGGGGAGAAGAA
AGAAAGAAAGAAAAAAAATTTTTAATTTCTCTTTTTGGTGTTGGTTAAAAGGGAACACAAGACATTTAAATAAAAC
ATCCCAAATATTTCTGAGGCC

Sequence Position	Motif	K-mer	Z-score	P-value
629	uukruuu	uuaauuu	2.813	2.45E-03
642	uukruuu	uuggugu	2.703	3.44E-03
648	uukruuu	uugguua	2.758	2.91E-03

>Mmp3

AAAGAGATCCAAGGAAGGCATCCTGTGTTTTAACTGATGCTTATAGTTCTTCATCTGAGTCTTTGTGAAAGGAAGT
GCTTTGTTCAGCATGTGCTATGGCAGAACCAAACAGGAGCTATGGATGACACCAGTCAACGTCAAGTTGTCAAAG
GATGTTCAGAAGCACTGTGTAGCTTACACTGTGTCCCAAGGAGAGGAGGGAAGGCACTCCTGGGCCACAAACAA
GTGTCTGAACTGTGTAGATGGTTTGTTTTATTTAATAAAGATTGTGTGTCGTTTATTAAA

Sequence Position	Motif	K-mer	Z-score	P-value
243	uukruuu	augguuu	3.593	1.63E-04
247	uukruuu	uuuguuu	3.67	1.21E-04
248	uukruuu	uuguuuu	3.714	1.02E-04
252	uukruuu	uuuauuu	3.692	1.11E-04

>Tnn

AGGCTTGTCTGAGCTGTCTTCACAGGAGACAAGAGAAACTTGGGGTTGGCGGGGTAGATGGTGCCTAGGAAGCT
GAAGTTTGAGGGATGCTCATAGCCTCCGACTTCCTAGATTGCTGGGTACACAGTGCTAATGACGTCATCACACTTT
GACCTTTAGAGGTTCCTTCCATGTCATCTGCAAGTTTGCCTGTCTCTACTGAGGGCTTCATTGTTGGCTTTCAGGAG
CCAACAATGTTGTAACTGAACAGTACATATGGATAGAACACGTGTTGGAATCACAAGGTTTTTCAGCCCATCCTTA
ATGACAGATGTATTGAATTAGGGCAAAAAGGTGAACCATCCTGTAGACCATCAATTGGAAATCTTTAGAAATTGGT
GGATATCTCTGTATCCATCTGATAATATAGATCTTTGTAGAAAAAAAAAGACAAAGTGAGGTTCTGATCCCCTGGG
TGTTAGAGCTGAGGTGACCTGCTACTTCTTGTCTCTCCCGGACCTCCTGGTGTTTGAAGGAAATGTTTATATCTTAC
TCCTTTTAAACTTCCTGTGTGATGAAGAATGAAGAGACTACAAAGGGGCCACGATGCAGTCATAGTTAAGTTCTGA
CTAGTCTCTAGCATTCTAGAGACCAAAGCTCCCATTTTATTGCTATTTTTAACTGCCCCTTTTCCAGACATTTGCATA
AGTTCTTTCATAGATCTGCATATGTTGTAAATAAATTTGCAGTCATTTAAACTTTAAATAACCCATCTCTCTAAATAA
CTGGTCCCCTCAGCTCCCCCCCACCACTTCACTCACCTCCATGCCTCTCCCCTCCCCCGGGTCCATCAGCCCTCAGAC
TCCCTCCTTCCCTGCTCCCCACACTGGAAGCTTCCTCACATCATCTTTCTTGCGTGTTTGGTGTCATTTTGGAAAGTTT
TCTGTAACCAAGTCAGTGACCTTTGAAGTAACTTAAATTCCTACTCAAGAAAACACAAATAAACTGTTTGATTTTTA
AAAA

Sequence Position	Motif	K-mer	Z-score	P-value
214	uukruuu	uuggcuu	2.275	1.15E-02

>Mmp13
GCATCTTTAAAAGTTGTTATTTATCTCCCAGAGAGTATTTGGAATACTTTCAGATGTATGGGGTGGGGGTGGGGTG
GAGATATCAGGGGAGAGCTTAGTTCTGTGAACGAGCTTCAGTAAGTTATCTTTGAGCATACAGTATCTATATGACT
ATGCGTGGCTGGAACCACATGGAAGAATTTTAAAGTAATGCAATTGAGAACCCCAAGGATCACCTGATTCTTGCGT
GCTATGAAGAAACAAGATTGATAATAACCCACAGCAAACATGGGGTCCATCTGCTTTTGAGAGCATGCATAATTAT
TAATATA**TTTATTTT**AAAAAGCCTAACAGACATAAAATAAATCATATTTATATAACTGAATTGTCTTTACAAAAAAG
TATAAACTTAGAAACTTGAAAATTGTGAGGAGTTCATGTATGGGGAGCCACAGATGAGCACAGATAAAGGGAAAT
GCCTAAAAAATGCACGTTAACGGACAACTTTCCAAAGAGAGATTTCAGCTTTTCACTGCGAGCGTTCAGATTTACA
TCCACTTTTATACAACCAATAAAAAAATACCAAAGTCACTAAAGAAAGGGGATAACAGCCACTACAAGGACAGTG
GAGGTGGCCTTACAT**TTGGCTTAATTT**TTATGTTGGTCATTACTCAAGGCTATGCACACTGGTAGAAGATATTGAG
AGAGAAATGGAGGAGATTTCTCTTTTTATTAAATATTTAGGCATTGAAAAGACCATAGTGTGAAAAGTCAAAATTG
CTATAAGATACGTAAGCAATGCCATAGCTTTTTCATGAATTATTTGACTATTTTAGAATAAAACTAATGTTTCAAC**CT**
TGTTTATCTACCCACTTGTTCTAATGACCTATAGACTCTTTGATACATAGTCTCTTTTCTAGTAACTTGTGTGACAGG
GGCTAAGGCAGAAATATTATGTAGAAGTAGATCCAGCTAAGACACAGCAAGCCAGAATAAAGACTGTGCCAGCT
GGTCAGTCGCCCTTTTGAGACCACTCCTTTGTGCTCCACCATGTTTGTTAATCCCTCTCTGCTTTCCTTAGCGAGTAA
CACTTGGTGCTTACTGATGTGTGAAAAGCTATTGTGTCAAGAGACAGTGTTAATTAAACTGGGAAAATACAAAAG
AACTG**TTTTTTT**GAATAATATGTTAGACTGTATTTAT**GTTGTTT**CTAATAAAAATAAGTGTTTTCAGCAGA

Sequence Position	Motif	K-mer	Z-score	P-value
312	uukruuu	uuuauuu	2.747	3.01E-03
313	uukruuu	uuauuuu	2.615	4.46E-03
623	uukruuu	uuggcuu	2.615	4.46E-03
628	uukruuu	uuaauuu	2.659	3.92E-03
836	uukruuu	cuuguuu	2.319	1.02E-02
1148	uukruuu	uuuuuuu	2.725	3.22E-03
1180	uukruuu	guuguuu	2.692	3.55E-03

>Adamts9
CTAAGGGGCTCTGAAGAGGAAGCCATAGGAGACGGATGAAGGATAGTAATTCAATACCTCCACCTTGAATTTGGG
TGCATCTGTGTGCGTGTGTGTGGATCTGTCTGCGCGTGTGTGTGTGTGTGTGTGTGTGTGTGTGAATGTGTGTG
TATATGTGTGTACATATGCATATATATAAATATATATTATATATATATATATATACAGGTTGAGCATCCCTTATTTAA
AATTTTAAGCTCTGAAATGCTCCAAATTCTGAAAACTTTTGAGTGCTAGCATGAGATCTCAAATGGTAAATTTCATG
CCATGAAACTTTGCTTCATACACAAATTTTTATAAGTACCATATAAAATTATATTTCTGCCATGCATGTAAAGTTTCC
TAAGAAACAGAAAGGAGACACACAACCCACATTCTTGGGCATGGCTTGCTCTCTTCCTGAGTTCCCTACTGCTTAA
ACCCTACGCTAAAATCTCCTAGTAAACTACAACTCCGTTCAAAAGGAAGAGGCATAAATTAATAGCATGTTTAGAT
GAGCCCCATCCCAAAAAGATCTCATCTTAAATATGAAAATATCGCCAAGTCTTCAAAATGTCTGAAATACACACTGA
TGGTCTCAAGCATTTCAATGAGGGATACTCAACCTGCGGAACACCCTAATGATGTAAAGTTTAATATTTATGTTTGA
AATTATTTATTGTGATGTAATATTTTTGTACGTAAA**ATGATTT**TATTATGACTGCCTGTGCATGATCCTCCCCCTTGC
AGTCAGACAGCTGCATTTTACGTACTCTACATTATATGTAGTATATGACAAAGTGACTCTTCGTTATCACGGTACAC
T**GTTGTTT**ACTTTTTACCTGGAAATGTGTTTTACTGTTACATTTTGAAAATTTTTGTAA**TTATTTTTTT**AACAACCTAG
CTGACATCAAGGTGCTATGTGGGTTGAATAAGGGTATTTTTGGCAATTGTGGGGACGTTTCATTAGCCAATGAGTA
AGTTAGCAACATCACAGGTTGTACCAACTGTTCATCATAGTCTTTATTCCGTTTCATGTGATCTCTGGGAAGAATTG

TGCTGCCTTGGTGCTAATATTATATGTTTTTAAAGTAATTGTCAGAGTCAAAACTGTAAACACTTAACAAAAGGGA
GCAAGAAATAGATTATCCCCAATGGATAAAATCACTTGCTAGAGTAAGACCTACTATTTACTCTTCTTTTTAGACCT
GTCTTTTTTTTTTTAATAGTAGCTGGACTCTTGTCTTACTGTTGTTGTTGGTAATATGATCTACTCTGCTCCATGGTG
CCTTGGACTCCTGTCAATCATTGGGAGGGTCCACCAAAAGCTTCATTACAGAGTGACACTGGGAAAGGAGGCTCT
CCTGCTGTTTTGTCACGATATCCTCCAAGTTTATGAGCAGCAACACAAAGGATCTACCACAGGTGTATGGCCTTAAC
ACCCCTCAGCATACTCAGAGAACGACAGGAGATCGACCACCTTTTACCTGAAAACTTTGGCCAATGATATGACTTC
CCTTGAGGCTGGGAGCCTAATACCCTGCCGGGTTTCCTGTGGTCTTCAGCGTTTGTGTGAAAAAACAAACTGGTGT
CTAAGATGCCCAGGTCTTTCTGATCTTCCACGAATCCACCTTTTGTTACTTCTTTCAGGAATGAACGTGCTGAGGCA
GTGGTCACAAGACATTTTACTTGAGAAAGCCTGACCAAGCTTTCCAAGACCAAAGAGATTAGGAAAACCCTGACA
GTATTCCCCAACTCCACAGAAATGCCAGAGCACTTGGTGTTTGAACAAGTGTCATTTTTCTATTGTTAATACATGTG
AAACATATTATAAATAAATATTGTGACCACATCCAAACC

Sequence Position	Motif	K-mer	Z-score	P-value
727	uukruuu	augauuu	2.407	8.04E-03
847	uukruuu	guuguuu	2.89	1.93E-03
905	uukruuu	uuauuuu	2.857	2.14E-03
908	uukruuu	uuuuuuu	2.923	1.73E-03
1234	uukruuu	uuuuuuu	3.33	4.34E-04
1235	uukruuu	uuuuuuu	3.33	4.34E-04
1236	uukruuu	uuuuuuu	3.341	4.17E-04
1237	uukruuu	uuuuuuu	3.341	4.17E-04
1238	uukruuu	uuuuuuu	3.341	4.17E-04
